# Adherence to the RSA and CT-RSA guideline items in clinical prosthesis migration studies: a systematic review

**DOI:** 10.2340/17453674.2025.43750

**Published:** 2025-05-27

**Authors:** Thies J N VAN DER LELIJ, Lennard A KOSTER, Bart L KAPTEIN, Rob G H H NELISSEN, Perla J MARANG-VAN DE MHEEN

**Affiliations:** 1 Department of Orthopaedics, Leiden University Medical Center, Leiden, Zuid-Holland; 2 Safety & Security Science and Centre for Safety in Healthcare, Delft University of Technology, Delft, Zuid-Holland, The Netherlands

## Abstract

**Background and purpose:**

Standardized reporting on methodology and results in clinical RSA research papers facilitates evaluation of quality and interpretation of results. We aimed to assess the extent to which radiostereometric analysis (RSA) and computed tomography-based RSA (CT-RSA) studies adhered to the items of the new RSA reporting guideline from 2024.

**Methods:**

A systematic literature search was performed to identify all clinical RSA studies published between January 2012 and February 2024. Studies were eligible for inclusion if prosthesis migration over time was assessed. The adherence of studies to each applicable guideline item (full, partial, or no) was assessed.

**Results:**

285 studies were included, most of which assessed prosthesis migration in the hip (n = 161) or knee (n = 99). No study reported on all guideline items. The mean (full or partial) adherence of studies to all (applicable) items was 61% (standard deviation [SD] 11). Large variation between the reporting of items was found, ranging from being reported in 1% of the studies to 100%. The least reported items in studies were the mean number and SD of days between surgery and baseline RSA examination (8% of studies), mean number and SD of days between surgery and primary endpoint RSA examination (1%), and consistent- or all-marker method for RSA analysis (3%).

**Conclusion:**

Current studies on average reported only 61% of the items from the updated RSA guidelines. Adherence to the guidelines in clinical RSA studies on prosthesis migration should be improved, in order to improve the quality of studies and the interpretation of outcomes on implant migration.

The reporting quality of radiostereometric analysis (RSA) studies has greatly improved since publication of the first RSA guidelines [1,2]. The guideline for standardization (2005) and the ISO standard for RSA (ISO 16087:2013) aimed to facilitate consistency in the execution, presentation, and interpretation of RSA studies [1]. High reporting quality is a prerequisite for assessing methodological quality of a study and thereby the article. It has been shown that the proportion of RSA studies with high reporting quality increased almost 3-fold in the period 2006 to 2011 compared with the period before the 2005 guideline [2,3]. Nevertheless, the overall adherence of clinical studies to guideline items remained relatively low [2].

Recently, updated guidelines for RSA and computed tomography RSA (CT-RSA) studies were published by a group of RSA researchers from the International Radiostereometry Society [4]. As migration assessment methods have been further developed and introduced in the past decade, there was a need to update the guidelines to make them better aligned with current standards [4-6]. A new reporting checklist with 32 items was presented to serve as a reference for prosthesis migration studies (Table 1) [4]. 10 items were already (partially) listed in standardized output for clinical RSA studies in the previous RSA guidelines (Table 2, see Appendix) [1]. The other 22 items, not previously included in the standardized output, are expected to be used in different RSA studies because the updated guidelines reflect the current RSA reporting standard by experts in the field.

**Table 1:** Checklist items for prosthesis migration studies as presented in the updated RSA and CT-RSA guidelines (adapted from Kaptein et al. 2024 [4])

Checklist item	Studies where item was applicable, n	
*Title and abstract*		
1.	Identification as a radiostereometric (RSA) study or CT-based radiostereometric (CT-RSA) study in the title	285
2.	Identification as a radiostereometric (RSA) study or CT-based radiostereometric (CT-RSA) study in the abstract and keywords	285
*Methods*		
3.	Report papers/references where prior results or partial results can be found (e.g., the 2-year results have been published previously)	285
4.	First and last inclusion (e.g., March 1998–December 2000)	285
5.	Country and hospital(s) where surgeries were performed	285
6.	Number of surgeons (and number of surgeries per surgeon) who performed the surgeries	285
7.	Detailed description of prosthesis, cement/coating, and liner characteristics for each study group	285
8.	Report whether the first postoperative examination was obtained before or after weightbearing	285
9.	Mean number and SD of days between surgery and the baseline RSA examination	285
10.	Mean number and SD of days between surgery and the primary endpoint RSA examination	285
11.	Migration measurement method (marker-based RSA, model-based RSA, CT-RSA)	285
12.	Patient position (supine, weightbearing)	
13.	Software used, including version number	285
14.	Location and orientation of the migration coordinate system	285
15.	Use of fictive/feature points to calculate MTPM	150
*Marker-/model-based RSA technique*		
16.	Image resolution (DPI) and type (CR, DR, film) of X-ray detectors	283
17.	Material and size of markers	283
18.	Calibration cage used, including type (uniplanar, bi-planar)	283
19.	Cut-off values for condition number and mean error of rigid body fitting	283
20.	Consistent- or all-marker method for RSA analysis	283
*CT-RSA technique*		
21.	CT-scanner brand and model	5
22.	Voxel size, slice thickness, kV, mAs	5
23.	Was metal artifact reduction used	5
24.	Effective radiation dose in mSV (for hip, spine, shoulder)	5
*Results*		
25.	Number of migration examinations for each study group and follow-up timepoint used in the primary analysis	285
26.	Number of and reasons why migration examinations (including double examinations) were missing or excluded; may also be reported in the methods	285
27.	All migration data should be presented in millimeters (translations) and degrees (rotations)	285
28.	Double examinations: mean, SD, and n for all outcome variables in the study (including 3 translations, 3 rotations, MTPM, TT, and TR if relevant) should be presented in a table for each study group separately	285
29.	Mean and SD of number of markers, condition number, and mean error of rigid-body fitting for each rigid body (bone/prosthesis) at the primary follow-up timepoint	285
30.	Unmodelled (raw data) of translation, rotation, and MTPM results: mean, n, and one of the following [CI, SD], or median and interquartile range for non-normal data for each study group and follow-up timepoint should be presented in a table or figure or both. If this table or figure does not fit in the manuscript, then it should be placed in supplementary data, or at least be available upon request	285
31.	Number of prosthesis revision/failures in each treatment group, including reason (e.g., revision due to aseptic loosening)	285
32.	Migration values at the last follow-up before revision or failure	176

**Table 2:** Standardized output for clinical RSA studies from the old RSA guidelines

1.	Units used for translation should always be millimeters and the units used for rotations should be degrees.
2.	Accuracy and precision of the arrangement used should be presented. Measurement interval and window tolerance should be quoted
3.	Type of calibration cage (object) and use of reference plates should be given
4.	It should be stated whether fixed or portable X-ray sources were used
5.	Positioning of subject, calibration cage (object), X-ray tubes, and X-ray cassettes should be standardized or described in detail. Orientation of the global coordinate system should be presented
6.	Method of image acquisition should be stated, e.g., whether scanned (then scanner details should be given) or whether digital radiographs have been used (then system details should be given)
7.	Software used should be stated, and if appropriate which version
8.	Size of marker beads used should be given (and validation results should be reported for the sizes used in the study)
9.	Method of determining the position of the implant, whether based on attached beads, geometrically, or model-based should be stated. If appropriate, reference to any new/novel technique should be given
10.	The following should be stated: cut-off level for condition number and rigid body fitting error for exclusion of subjects from study
11.	Rigid body fixed coordinate frames and angular rotation sequence should be defined
12.	Precision of the measurements assessed by double examinations of all patients enrolled in the study should be stated
13.	Migration/motion data should be given in terms of translations and angular rotations. All 6 degrees of freedom should be reported. If not, these data should be available from the authors on request. The point(s) used to measure translations should be indicated (either a single point of a rigid body or the center of gravity of a rigid body), standardized, and its (their) location(s) on the implant (or in the bone) should always be presented

Adapted from: Valstar et al. Guidelines for standardization of radiostereometry (RSA) of implants. Acta Orthop 2005; 76(4): 563-72.

The aim of this study was to assess to what extent RSA studies on prosthesis migration adhered to items presented in the updated RSA guidelines. Examining adherence and particularly those items frequently not reported may encourage researchers of future studies to improve the reporting quality of RSA studies and thereby their clinical value in the safe introduction of new implants.

## Methods

### Study design

This is a systematic review reported in accordance with the Preferred Reporting Items for Systematic Reviews and Meta-Analyses (PRISMA) statement [7].

### Search, screening, and selection

A systematic literature search was constructed in collaboration with an experienced clinical librarian (JS). PubMed, Cochrane Library, Embase, Emcare, Academic Search Premier, and Web of Science were searched to identify all publications between January 2012 and February 2024, as Madanat et al. [2] have already assessed adherence among studies published up to December 2011. The search was composed of the components “RSA” and “Prosthesis” (see Supplementary data). No term for specific joints (e.g., hip, knee, or shoulder) was added, as the guidelines are not specific for the type of joint or prosthesis assessed in the studies. After removal of duplicate studies, title and abstract screening was performed independently by 2 reviewers (TJNvdL and LAK). Subsequently, the full-text screening was independently performed by the same 2 reviewers and any disagreements were resolved through discussion.

Studies were eligible for inclusion if prosthesis migration relative to its surrounding bone was assessed in humans in vivo over time using RSA. There were no restrictions regarding the RSA method used (marker-based RSA, model-based RSA, CT-RSA), study design (randomized controlled trial [RCT], cohort study, case series), sample size, or follow-up time. Articles in English, Dutch, German, and French were considered and translated if necessary. Studies were excluded if only wear (of the polyethylene components) or inducible displacement (i.e., displacement occurring instantaneously as a result of an external load such as weightbearing) was assessed.

### Data extraction

Data was extracted independently by 2 reviewers (TJNvdL and LAK) using a prespecified SPSS file (IBM SPSS Statistics 29.0; IBM Corp, Armonk, NY, USA). For each study, the first author’s name, title, year of publication, journal, country in which the study was performed, study design, number of included patients, type of arthroplasty, type of RSA method, and duration of follow-up were extracted. We assessed whether prosthesis migration was the primary or secondary outcome of the study. Additionally, it was determined whether original migration data was presented or whether a reanalysis of previously published migration data was performed. Both reviewers evaluated each study for its adherence to the reporting checklist as presented in the updated RSA guidelines. Only applicable items were evaluated for each study. For example, a study using marker-based or model-based RSA does not need to adhere to items 21 to 24, as these are only relevant for CT-RSA studies (see Table 1). Also, when no maximum total point motion (MTPM) is calculated, a study will not report whether fictive/feature points are used (item 15). If there were no revisions in a study, migration values at the last follow-up before revision can also not be given (item 32). 21 items could be scored as full, partial (reporting at least 1 issue/sub-item), or no adherence. 11 items could only be scored as full or no adherence. Consensus was reached between the reviewers through discussion if different information was extracted or in case of disagreement in scoring items.

### Ethics, registration, data sharing, use AI-tools, funding, and disclosure

No ethical approval was required for this study as the data was retrieved from previously published studies. A protocol for this systematic review was registered with PROSPERO prior to screening of studies (ID: CRD42024540186). AI tools were not used. No funding was acquired for the present review and the authors declare no competing interest. Complete disclosure of interest forms according to ICMJE are available on the article page, doi: 10.2340/17453674.2025.43750

## Results

### Literature search and study selection

**Figure 1:**
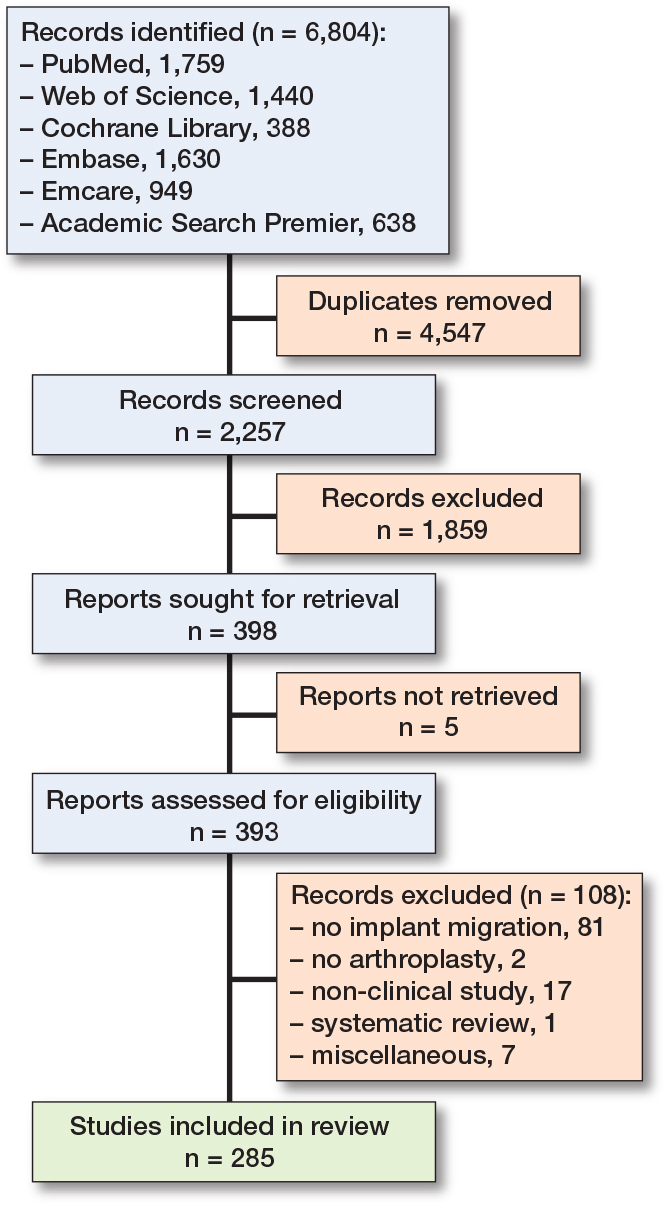
PRISMA flow diagram

Our literature search identified 2,257 unique records that were screened for eligibility (Figure 1). After title and abstract screening, 1,859 studies were excluded. Following the exclusion of 108 studies based on the full text and 5 reports that could not be retrieved, 285 eligible studies were included (see Supplementary data for reference list of articles).

### Characteristics of included studies

**Figure 2:**
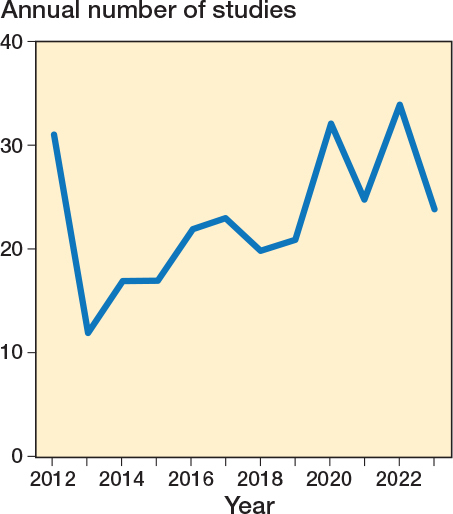
The annual number of clinical RSA studies on prosthesis migration.

The majority (n = 194) of the studies were performed in Denmark, Finland, The Netherlands, Norway, and Sweden (Table 3). Most studies were published in Acta Orthopaedica (n = 77), the Bone & Joint Journal (n = 53), and the Journal of Arthroplasty (n = 28). The annual number of published RSA studies showed an increasing trend in the last decade after a slight drop in 2013 (Figure 2). Prosthesis migration over time was the primary outcome of 244 studies, compared with 41 studies that assessed migration as a secondary outcome. 260 studies presented original clinical migration data, whereas 25 studies performed a reanalysis of previously published implant migration data. 149 RCTs, 135 cohort studies or case series, and 1 case report were included. Model-based RSA was applied in most studies (n = 158), followed by marker-based RSA (n = 128) (Table 3). The median sample size was 47 patients (interquartile range [IQR] 29–61) with a median follow-up time of 2 years (IQR 2–5). Various prosthesis components were assessed, including tibial (n = 96) and femoral (n =17) components in the knee, and acetabular (n = 60) and femoral (n = 106) components in the hip (Table 4).

**Table 3:** Characteristics of all clinical RSA studies on prosthesis migration published between 2012 and 2024

	Number of studies
Country of study	
NOF countries^a^	194
Rest of Europe^b^	29
Northern America	44
Australia	18
Journal of publication	
Acta Orthopaedica	77
The Bone & Joint Journal	53
The Journal of Arthroplasty	28
The Journal of Bone & Joint Surgery	16
Knee Surgery, Sports Traumatology, Arthroscopy	10
Hip International	22
Other	79
Type of RSA method used^c^	
Marker-based RSA	128
Model-based RSA	158
CT-based RSA	5
Non-specified	9

NOF = Nordic Orthopaedic Federation.

a Denmark, Finland, The Netherlands, Norway, and Sweden.

b Croatia, Germany, Italy, United Kingdom, and Switzerland.

c Some studies applied 2 RSA methods.

**Table 4:** Prosthesis components of which migration was assessed in clinical RSA studies

Joint	Component	Number of studies
Knee	Tibial	96
	Femoral	17
Hip	Acetabular	60
	Femoral	106
Ankle/foot	Tibial (talocrural joint)	1
	Talar (talocrural joint)	1
	Proximal phalanx (MTP-1 joint)	1
Shoulder	Glenoid	9
	Humeral	7
Elbow	Humeral	2
Wrist/hand	Radial (radiocarpal joint)	1
	Carpal (radiocarpal joint)	1
	Trapezoid (CMC-1 joint)	3
Spine	Superior vertebral (cervical spine)	1
	Inferior vertebral (cervical spine)	1

The total number of studies exceeds 285, as some studies assessed the migration of multiple components.

### Adherence of studies to updated RSA guidelines

**Figure 3:**
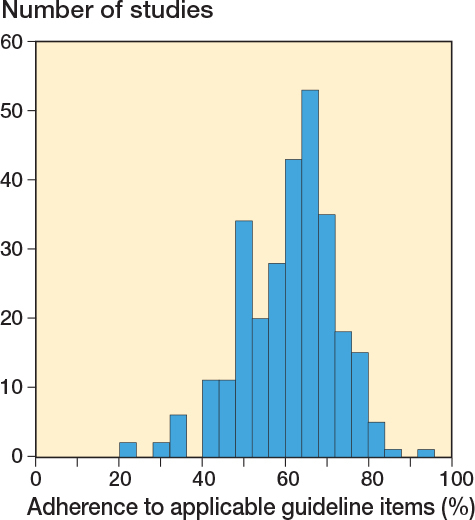
Adherence (full or partial) of RSA studies to the guideline items (when items were applicable).

We retrospectively applied the updated RSA guidelines to the included studies and none of them reported all items from the updated RSA guidelines. Studies adhered (fully or partially) to a mean of 61% (standard deviation [SD] 11%) of all applicable guideline items (Figure 3). The study with the highest adherence reported 92% of all applicable items (fully or partially). The study with the lowest adherence reported only 22% of the applicable items. 51 studies reported 50% or fewer of the guideline items. When considering only full adherence (not partial) to the checklist items, the study with the greatest adherence completely reported 65% of all applicable items. The study with the lowest adherence fully adhered to only 1 item of the guideline (4%).

**Figure 4:**
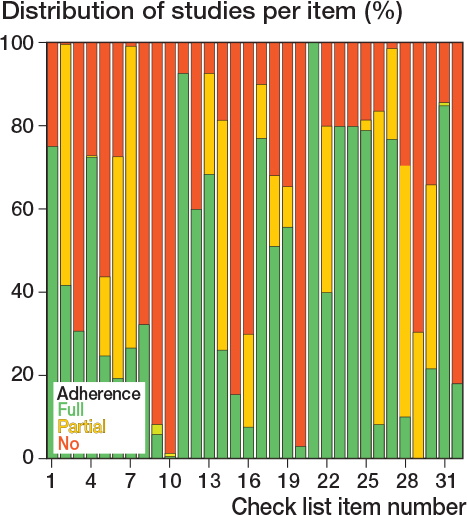
Adherence of RSA studies to the guideline items (when items were applicable).

The items most frequently reported (fully or partially) were items 2 (identification of RSA in abstract and keywords; reported by 100% of studies for which it was applicable), 7 (description of prosthesis; 99% of studies for which it was applicable), 11 (RSA method; 93% of studies for which it was applicable), 24 (CT-scanner brand and model; 100% of studies for which it was applicable), and 27 (migration data in millimeters and degrees; 99% of studies for which it was applicable) (Figure 4). Still, a considerable number of studies did not adhere fully to these items but only partially. For example, most studies reported the type of prosthesis used but without a detailed description of all components (e.g., liner characteristics, such as highly cross-linked polyethylene or vitamin E infused) (item 7). Also, most studies could not fully adhere to item 2 (identification of RSA in abstract and keywords), as some journals do not present keywords in the full text paper.

Items 9 (days to baseline RSA), 10 (days to primary endpoint RSA), and 20 (consistent- or all-marker method) were only reported (fully or partially) in, respectively, 8%, 1%, and 3% of the studies. There was 1 item (item 29; markers, condition number, and mean error) to which no study fully adhered (see Figure 4). To fully adhere to item 29, the mean and SD of both the number of markers, condition number, and mean error of rigid-body fitting for each rigid body (bone/prosthesis) at the primary follow-up timepoints needs to be reported.

## Discussion

We aimed to assess the extent to which RSA and CT-RSA studies adhered to the items of the new RSA reporting guideline from 2024. During the last decade, RSA studies on prosthesis migration reported only 61% of the recently published RSA guideline checklist items. Moreover, large variation between studies existed and some items were rarely reported. The present review provides an overview of current practice and offers directions on where the reporting quality of RSA can be improved. Although all studies were published before publication of the new guideline (May 2024), the guidelines can be considered to reflect the current reporting standard in the field of RSA, based on the opinion of expert swho may also have acted as reviewers for RSA studies and thereby signaled missing information, so we may expect that studies included in this review would adhere to (most of) the items. Moreover, the items presented in the RSA guideline should be viewed as a minimum and authors are encouraged to provide additional information when deemed necessary (4).

Nevertheless, not all guideline items can be applied in every study; however, this is not always clear for specific items. For example, when a study does not report where prior or partial results can be found (item 3), the reader may be left uncertain as to whether such results do not exist or whether the authors simply failed to report their location. To enhance the utility and practical implantation of the RSA guideline in future clinical studies, we propose some clarifications of the RSA guideline checklist (Table 5). In this respect, item 32 states that migration values at the last follow-up before revision or failure need to be reported, but does not specify whether this should be the mean migration of the revised implants or the complete study group or individual implant migration of the revised implants. For clarity, mean migration of the revised and non-revised group as well as individual implant migration of revised implants should be given. As for the last 2 checklist items (31 and 32), the definition of “failure” of a prosthesis is ambiguous and may differ between studies. Thus, a clear definition of “failure” should be given in the text. As for item 29, which is only relevant when marker- or model-based RSA is used, this should be part of the “methods RSA technique” section (see Tables 1 and 5).Ittem 25 states that the number of migration examinations for each study group and follow-up timepoint used in the primary analysis should be presented. However, in clinical RSA studies it is often unclear what constitutes the “primary analysis.” In the context of clinical trials, the primary analysis refers to the analysis prespecified in the protocol that will answer the main research question, mostly using an intention-to-treat approach, whilst a secondary analysis can use an as-treated approach. When the migration up to 2-year follow-up is reported as the primary outcome, this means that the migration results of all previous follow-up moments are also included in the analysis and therefore that it holds merit to report the included examinations at all timepoints included in the analysis. Using a complete study flow diagram would solve this problem by showing both the number of patients and included examinations for each group at all different follow-up timepoints. Such a flow diagram can also be used to adequately report the number of and reason why migration examinations were missing (item 26).

**Table 5:** Proposal for updated checklist for prosthesis migration studies

Checklist item
*Title and abstract* Identification as a radiostereometric (RSA) study or CT-based radiostereometric (CT-RSA) study in the title. Identification as a radiostereometric (RSA) study or CT-based radiostereometric (CT-RSA) study in the abstract (and keywords **if available***).*
*Methods* Report papers/references where prior results or partial results can be found (e.g., the 2-year results have been published previously) (**if applicable**). First and last inclusion **date of surgery of included patients** (e.g., March 1998–December 2000). Country and hospital(s) where surgeries were performed. Number of surgeons (and number of surgeries per surgeon **in each study group**) that performed the surgeries. Detailed description of **all components of the** prosthesis, **including** cement/coating, and liner characteristics for each study group. Report whether the first postoperative examination was obtained before or after weightbearing (**for joints of the lower extremities or spine**). Mean number and SD, **or median and IQR**, of days between surgery and the baseline RSA examination. Mean number and SD, **or median and IQR**, of days, **weeks, months or years** between surgery and the primary endpoint RSA examination. Migration measurement method (marker-based RSA, model-based RSA, CT-RSA) Patient position (supine, weightbearing) **during all follow-up examinations.** Software used, including version number. Location and orientation of the migration coordinate system. Use of fictive/feature points to calculate MTPM (**if applicable**).
*Marker-/model-based RSA techniqu*e Image resolution (DPI) and type (CR, DR, film) of X-ray detectors. Material and size of markers. Calibration cage used, including type (uniplanar, bi-planar). - Cut-off values for condition number and mean error of rigid body fitting. **Mean and SD of number of markers, condition number, and mean error of rigid-body fitting for each rigid body (bone/prosthesis) at the primary follow-up timepoint.** Consistent- or all-marker method for RSA analysis.
*CT-RSA technique* CT-scanner brand and model. Voxel size, slice thickness, kV, mAs. Was metal artifact reduction used. Effective radiation dose in mSV (for hip, spine, shoulder).
*Results* Number of migration examinations for each study group and follow-up timepoints used in the primary analysis. Number and reasons why migration examinations (including double examinations) where missing or excluded **at each timepoint for each study group**; may also be reported in the methods. All migration data should be presented in millimeters (translations) and degree (rotations). Double examinations: mean, SD, and n for all outcome variables in the study (including 3 translations, 3 rotations, MTPM, TT, and TR if relevant) should be presented in a table for each study group separately. Mean and SD of number of markers, condition number, and mean error of rigid-body fitting for each rigid body (bone/prosthesis) at the primary follow-up timepoint. Unmodelled (raw data) of translation, rotation, and MTPM results: mean, n, and one of the following [CI, SD], or median and interquartile range for non-normal data for each study group and **all** follow-up timepoints should be presented in a table or figure or both. If this table or figure does not fit in the manuscript, then it should be placed in supplementary data, or at least be available upon request. Number of prosthesies revision**s**/failures in each treatment group, including reason (e.g., revision due to aseptic loosening). **If revisions occurred, provide** migration values at the last follow-up before revision **for each revised prosthesis individually** or failure.

Proposed changes to the checklist items are in bold (addition) or as strikethrough (removal)

An explanation for the moderate adherence of clinical RSA studies to the checklist items could be the strict formulation and interpretation of listed items. For example, 2 of the least reported items (9 and 10) state that the mean and SD of days between surgery and both baseline RSA examination and primary RSA endpoint need to be reported. Some studies provided the median and IQR as alternative measures of the variation, which provides relevant information on the distribution, and are preferred for data with a non-normal distribution (see Figure 4).

Authors may miss some of the recommendations that were described in the text of the guideline paper if they focus only on the checklist of the new RSA guideline. For example, in the text of the updated guidelines it is described that a consistent set of fictive points to report MTPM is advised for marker-based RSA and CT-RSA, but not for model-based RSA (even though the reason for this remains unclear considering the fact that both CT-RSA and model-based RSA use prosthesis models with a large number of points on the outer surface). However, the reporting checklist does not restrict the use of fictive/feature points to calculate MTPM to specific RSA methods. According to the checklist, all studies should report the use of fictive/feature points to calculate MTPM, regardless of RSA method, which may explain the relatively low adherence to item 15. As for item 30, the checklist states that unmodelled (raw) data of translation, rotation, and MTPM results should be presented. However, in the text of the guidelines it is advised to use suitable statistical analysis techniques such as (generalized) linear mixed models (LMM) to analyze the results. When only unmodeled data has to be presented, this may give biased mean results for each study group as missing data and correlations between measurements of the same patients are not accounted for.

The least reported item was the use of the consistent- or all-marker method (item 20). A recent paper drew attention to the issue of different marker-selection methods and their influence on migration results, so that reporting of this item may improve in future studies [8]. Finally, although the migration measurement method (item 11) was frequently reported in studies, we wish to draw attention to some of the implicit assumptions being made (see Figure 4). If a study merely describes that markers were attached to the prosthesis, this does not automatically indicate that marker-based RSA was used for the migration analysis, as it is still possible to perform migration analysis with model-based RSA. Furthermore, the name of the software “Model-Based RSA (RSAcore, Leiden, The Netherlands)” may be confusing for readers not familiar with RSA, as both marker- and model-based RSA analysis can be performed with this software. Therefore, the RSA method used for analysis should be explicitly reported.

Madanat et al. [2] previously reported that nearly half of the studies published between 2006 and 2011 adhered at least partially to 10 of the 13 old RSA guideline items published in 2005, whereas this was less than one-fifth of the studies published between 2000 and 2005. Therefore, it might be expected that adherence to the updated guideline items will also increase following publication of the new guideline in 2024. However, Madanat et al. [2] also found that even after publication of the old RSA guidelines in 2005, none of the studies fully met all guideline items. The latter underlines the importance of the present review, as we highlight topics that are frequently missing to promote their reporting in future studies.

### Limitations

First, we searched only for clinical studies using RSA to assess implant migration. However, studies using other methods to assess implant migration exist, such as Ein Bild Roentgen Analysis (EBRA) and CT-based implant migration analysis without the term RSA, which may have been missed by our literature search [9-11]. For implant migration studies that do not include the term RSA, we expect that these authors are not aware of the RSA method and guidelines. Second, we assessed the reporting quality of studies performed between 2012 and 2024, before publication of the updated RSA guidelines in 2024. However, as the updated RSA guidelines represent current practice, we expected the papers to generally adhere to the items. Third, follow-up studies may reference papers with prior results, which may give a more detailed description of the methods, but items from these previous studies were not accounted for in the evaluation of the follow-up studies. However, in the opening remarks of the updated RSA guideline it is stated that deviations from these guidelines should have the underlying rationale stated. This provides researchers with the flexibility to not report all items and assists in the practical implementation of the updated guidelines.

### Conclusion

Clinical RSA studies on prosthesis migration on average reported only 61% of the items presented in the recently published RSA guidelines.

*In perspective,* our results can be used by RSA researchers and clinicians to guide interpretation of items and highlight the importance of complete reporting to improve the reporting quality for future studies. Furthermore, we argue that rewording of specific checklist items may also contribute to increased adherence of clinical RSA studies to the updated RSA guidelines. Further, we urge the reviewers of RSA manuscripts to ask for the reporting checklist and that this should be available as supplementary material as for any other reporting guideline.

### Supplementary data

A reference list of all 285 included articles is available as supplementary data on the article page, doi: 10.2340/17453674.2025.43750

## Supplementary Material





## Notes

TvdL: conceptualization, methodology, investigation, data curation, visualization, writing—original draft. LK: methodology, investigation, writing—review and editing. BK: methodology, supervision, writing – review and editing. RN: methodology, writing—review & editing. PM: conceptualization, methodology, supervision, writing—review and editing.

The authors would like to thank Jan Schoones (JS), clinical librarian, for his help with performing the literature search.

Handling co-editor: Per Henrik Randsborg

*Acta* thanks Cyrus Brodén and Lene Bergendal Solberg for help with peer review of this manuscript.
