# Supplementary material for: Adherence to the RSA and CT-RSA guideline items in clinical prosthesis migration studies: a systematic review
Source: Acta Orthop. 2025 May 27;96:380–6. doi: 10.2340/17453674.2025.43750 (PMC12118374; doi:10.2340/17453674.2025.43750)
Supplement: Supplementary file 1 [file ActaO-96-43750-s1.pdf]

Appendix 3. References of all included studies (N = 285)

1. Abrahams JM, Callary SA, Jang SW, Hewitt J, Howie DW, Solomon LB. Accuracy of EBRA-cup measurements after reconstruction of severe acetabular defects at revision THR. J Orthop Res 2020; 38(7): 1497-505. doi: 10.1002/jor.24623.

2. Acklin YP, Jenni R, Bereiter H, Thalmann C, Stoffel K. Prospective clinical and radiostereometric analysis of the Fitmore short-stem total hip arthroplasty. Arch Orthop Trauma Surg 2016; 136(2): 277-84. doi: 10.1007/s00402-015-2401-9.

3. Akhtar A, Ricks M, Cunningham L, Moffatt M, Bale S, Walton M, et al. A randomized prospective study comparing migration of hydroxyapatite and non-hydroxyapatite coated glenoid components using radiostereometric analysis. Seminars in Arthroplasty JSES 2021; 31(4): 635-43. doi: https: //dx.doi.org/10.1053/j.sart.2021.04.001.

4. Alsousou J, Oragui E, Martin A, Strickland L, Newman S, Kendrick B, et al. Primary stability of a proximally coated and tapered stem. Bone Joint J 2021; 103-b(4): 644-9. doi: 10.1302/0301-620x.103b4.Bjj-2020-1648.R1.

5. Andersen MR, Winther N, Lind T, SchrøDer H, Flivik G, Petersen MM. Monoblock versus modular polyethylene insert in uncemented total knee arthroplasty. Acta Orthop 2016; 87(6): 607-14. doi: 10.1080/17453674.2016.1233654.

6. Andersen MR, Winther N, Lind T, Schrøder H, Flivik G, Petersen MM. Tibial Component Undersizing Is Related to High Degrees of Implant Migration Following Cementless Total Knee Arthroplasty: A Study of Radiostereometric Analysis Data for 111 Patients with 2-Year Follow-up. JBJS Open Access 2023; 8(3). doi: 10.2106/jbjs.Oa.23.00032.

7. Andersen MR, Winther NS, Lind T, Schrøder HM, Flivik G, Petersen MM. Low Preoperative BMD Is Related to High Migration of Tibia Components in Uncemented TKA-92 Patients in a Combined DEXA and RSA Study With 2-Year Follow-Up. J Arthroplasty 2017; 32(7): 2141-6. doi: 10.1016/j.arth.2017.02.032.

8. Angelomenos V, Mohaddes M, Itayem R, Shareghi B. Precision of low-dose CT-based micromotion analysis technique for the assessment of early acetabular cup migration compared with gold standard RSA: a prospective study of 30 patients up to 1 year. Acta Orthop 2022; 93: 459-65. doi: 10.2340/17453674.2022.2528.

9. Aro E, Alm JJ, Moritz N, Mattila K, Aro HT. Good stability of a cementless, anatomically designed femoral stem in aging women: a 9-year RSA study of 32 patients. Acta Orthop 2018; 89(5): 490-5. doi: 10.1080/17453674.2018.1490985.

10. Aro E, Moritz N, Mattila K, Aro HT. A long-lasting bisphosphonate partially protects periprosthetic bone, but does not enhance initial stability of uncemented femoral stems: A randomized placebo-controlled trial of women undergoing total hip arthroplasty. J Biomech 2018; 75: 35-45. doi: 10.1016/j.jbiomech.2018.04.041.

11. Aro HT, Alm JJ, Moritz N, Mäkinen TJ, Lankinen P. Low BMD affects initial stability and delays stem osseointegration in cementless total hip arthroplasty in women: a 2-year RSA study of 39 patients. Acta Orthop 2012; 83(2): 107-14. doi: 10.3109/17453674.2012.678798.

12. Aro HT, Engelke K, Mattila K, Löyttyniemi E. Volumetric Bone Mineral Density in Cementless Total Hip Arthroplasty in Postmenopausal Women: Effects on Primary Femoral Stem Stability and Clinical Recovery. J Bone Joint Surg Am 2021; 103(12): 1072-82. doi: 10.2106/jbjs.20.01614.

13. Aro HT, Nazari-Farsani S, Vuopio M, Löyttyniemi E, Mattila K. Effect of Denosumab on Femoral Periprosthetic BMD and Early Femoral Stem Subsidence in Postmenopausal Women Undergoing Cementless Total Hip Arthroplasty. JBMR Plus 2019; 3(10): e10217. doi: 10.1002/jbm4.10217.

14. Ayers DC, Greene M, Snyder B, Aubin M, Drew J, Bragdon C. Radiostereometric analysis study of tantalum compared with titanium acetabular cups and highly cross-linked compared with conventional liners in young patients undergoing total hip replacement. J Bone Joint Surg Am 2015; 97(8): 627-34. doi: 10.2106/jbjs.N.00605.

15. Balesar VV, Koster LA, Kaptein BL, Keizer SB. Five-Year Prospective Roentgen Stereophotogrammetric and Clinical Outcomes of the BioPro MTP-1 Hemiarthroplasty. Foot Ankle Int 2022; 43(5): 637-45. doi: 10.1177/10711007211061366.

16. Barbadoro P, Ensini A, Leardini A, d'Amato M, Feliciangeli A, Timoncini A, et al. Tibial component alignment and risk of loosening in unicompartmental knee arthroplasty: a radiographic and radiostereometric study. Knee Surg Sports Traumatol Arthrosc 2014; 22(12): 3157-62. doi: 10.1007/s00167-014-3147-6.

17. Belfrage O, Tägil M, Sundberg M, Kesteris U, Flivik G. Locally administered bisphosphonate in hip stem revisions using the bone impaction grafting technique: a randomised, placebo-controlled study with DXA and five-year RSA follow-up. Hip Int 2019; 29(1): 26-34. doi: 10.1177/1120700018781809.

18. Bergvinsson H, Sundberg M, Flivik G. Polyethylene wear with ceramic and metal femoral heads at 5 years: a randomized controlled trial with radiostereometric analysis. J Arthroplasty 2020; 35(12): 3769-76. doi: 10.1016/j.arth.2020.06.057.

19. Bergvinsson H, Zampelis V, Sundberg M, Flivik G. Highly cross-linked polyethylene still outperforms conventional polyethylene in THA: 10-year RSA results. Acta Orthop 2021; 92(5): 568-74. doi: 10.1080/17453674.2021.1932140.

20. Bergvinsson H, Zampelis V, Sundberg M, Tjörnstrand J, Flivik G. Vitamin E infused highly cross-linked cemented cups in total hip arthroplasty show good wear pattern and stabilize satisfactorily: a randomized, controlled RSA trial with 5-year follow-up. Acta Orthop 2022; 93: 249-55. doi: 10.2340/17453674.2022.1517.

21. Bohm E, Petrak M, Gascoyne T, Turgeon T. The effect of adding tobramycin to Simplex P cement on femoral stem micromotion as measured by radiostereometric analysis: a 2-year randomized controlled trial. Acta Orthop 2012; 83(2): 115-20. doi: 10.3109/17453674.2011.652885.

22. Breddam Mosegaard S, Jørgensen PB, Storgaard Jakobsen S, Daugaard H, Søballe K, Stilling M. Larger 5-year migration but similar polyethylene wear of cementless hemispherical cups with electrochemically applied hydroxyapatite (BoneMaster) coating compared with porous plasma-spray titanium: a randomized 5-year RSA study. Acta Orthop 2022; 93: 658-64. doi: 10.2340/17453674.2022.3976.

23. Breddam Mosegaard S, Rytter S, Madsen F, Odgaard A, Søballe K, Stilling M. Two-year fixation and ten-year clinical outcomes of total knee arthroplasty inserted with normal-curing bone cement and slow-curing bone cement: A randomized controlled trial in 54 patients. Knee 2021; 33: 110-24. doi: 10.1016/j.knee.2021.08.027.

24. Brinke BT, Kosse NM, Flikweert PE, van der Pluijm M, Eygendaal D. Long-term outcomes after Instrumented Bone Preserving total elbow arthroplasty: a radiostereometric study with a minimum follow-up of 10 years. J Shoulder Elbow Surg 2020; 29(1): 126-31. doi: 10.1016/j.jse.2019.07.023.

25. Broberg JS, Koff MF, Howard JL, Lanting BA, Potter HG, Teeter MG. A multimodal assessment of cementless tibial baseplate fixation using radiography, radiostereometric analysis, and magnetic resonance imaging. J Orthop Res 2024; 42(1): 100-8. doi: 10.1002/jor.25662.

26. Broberg JS, Naudie DDR, Howard JL, Lanting BA, Vasarhelyi EM, Teeter MG. Correlating Contact Kinematics to Tibial Component Migration Following Cemented Bicruciate Stabilized Total Knee Arthroplasty. J Arthroplasty 2023; 38(6s): S355-s62. doi: 10.1016/j.arth.2023.01.051.

27. Broberg JS, Naudie DDR, Lanting BA, Howard JL, Vasarhelyi EM, Teeter MG. Patient and Implant Performance of Satisfied and Dissatisfied Total Knee Arthroplasty Patients. J Arthroplasty 2022; 37(6s): S98-s104. doi: 10.1016/j.arth.2021.10.024.

28. Broberg JS, Vasarhelyi EM, Lanting BA, Howard JL, Teeter MG, Naudie DDR. Migration and Inducible Displacement of the Bicruciate-Stabilized Total Knee Arthroplasty: A Randomized Controlled Trial of Gap Balancing and Measured Resection Techniques. J Arthroplasty 2022; 37(2): 252-8. doi: 10.1016/j.arth.2021.10.010.

29. Brodén C, Reilly P, Khanna M, Popat R, Olivecrona H, Griffiths D, et al. CT-based micromotion analysis method can assess early implant migration and development of radiolucent lines in cemented glenoid components: a clinical feasibility study. Acta Orthop 2022; 93: 277-83. doi: 10.2340/17453674.2022.1976.

30. Bruni D, Bragonzoni L, Gagliardi M, Bontempi M, Akkawi I, Raspugli GF, et al. Roentgen stereophotogrammetric analysis: an effective tool to predict implant survival after an all-poly unicompartmental knee arthroplasty-a 10 year follow-up study. Knee Surg Sports Traumatol Arthrosc 2015; 23(11): 3273-80. doi: 10.1007/s00167-014-3106-2.

31. Budde S, Derksen A, Hurschler C, Fennema P, Windhagen H, Plagge J, et al. Very early migration of a calcar-guided short stem: a randomized study of early mobilization and the influence of a calcium phosphate coating with 60 patients. Sci Rep 2024; 14(1): 3837. doi: 10.1038/s41598-023-50829-3.

32. Budde S, Seehaus F, Schwarze M, Hurschler C, Floerkemeier T, Windhagen H, et al. Analysis of migration of the Nanos® short-stem hip implant within two years after surgery. Int Orthop 2016; 40(8): 1607-14. doi: 10.1007/s00264-015-2999-9.

33. Bunting AC, Costi K, Chimutengwende-Gordon M, Callary SA, Pannach S, Nelson R, et al. Staged Revision Hip Arthroplasty With Femoral Impaction Bone Grafting for Prosthetic Joint Infections: Radiostereometric Analyses and Clinical Outcomes at Minimum 5-Year Follow-Up. J Arthroplasty 2023; 38(12): 2716-23.e1. doi: 10.1016/j.arth.2023.06.003.

34. Callary SA, Campbell DG, Mercer GE, Nilsson KG, Field JR. The 6-year migration characteristics of a hydroxyapatite-coated femoral stem: a radiostereometric analysis study. J Arthroplasty 2012; 27(7): 1344-8.e1. doi: 10.1016/j.arth.2011.12.002.

35. Campbell D, Callary S, Field J, Nilsson KG. All-polyethylene tibial components in young patients have stable fixation; a comparison RSA study. Knee 2019; 26(2): 392-9. doi: 10.1016/j.knee.2018.12.003.

36. Campi S, Kendrick BJL, Kaptein BL, Valstar ER, Jackson WFM, Dodd CAF, et al. Five-year results of a randomised controlled trial comparing cemented and cementless Oxford unicompartmental knee replacement using radiostereometric analysis. Knee 2021; 28: 383-90. doi: 10.1016/j.knee.2020.09.003.

37. Christensson A, Nemati HM, Flivik G. Comparison between model-based RSA and an AI-based CT-RSA: an accuracy study of 30 patients. Acta Orthop 2024; 95: 39-46. doi: 10.2340/17453674.2024.35749.

38. Christensson A, Tveit M, Kesteris U, Flivik G. Similar migration for medial congruent and cruciate-retaining tibial components in an anatomic TKA system: a randomized controlled trial of 60 patients followed with RSA for 2 years. Acta Orthop 2022; 93: 68-74. doi: 10.1080/17453674.2021.1983709.

39. Christiansen JD, Ejaz A, Nielsen PT, Laursen M. An Ultra-Short Femoral Neck-Preserving Hip Prosthesis: A 2-Year Follow-up Study with Radiostereometric Analysis and Dual X-Ray Absorptiometry in a Stepwise Introduction. J Bone Joint Surg Am 2020; 102(2): 128-36. doi: 10.2106/jbjs.19.00104.

40. Coffey SP, Sorial RM, Sharma R, Field JR. Two-year migration characteristics of a novel cementless femoral stem: a radiostereometric analysis and clinical outcomes study. ANZ J Surg 2021; 91(3): 398-403. doi: 10.1111/ans.16616.

41. Critchley O, Callary S, Mercer G, Campbell D, Wilson C. Long-term migration characteristics of the Corail hydroxyapatite-coated femoral stem: a 14-year radiostereometric analysis follow-up study. Arch Orthop Trauma Surg 2020; 140(1): 121-7. doi: 10.1007/s00402-019-03291-8.

42. Cunningham LJ, Walton M, Bale S, Trail IA. A prospective radiostereometric analysis of the stability of a metal-backed glenoid component/autograft composite in reverse shoulder arthroplasty. Bone Joint J 2023; 105-b(8): 912-9. doi: 10.1302/0301-620x.105b8.Bjj-2022-1280.R2.

43. Dahl J, Snorrason F, Nordsletten L, Röhrl SM. More than 50% reduction of wear in polyethylene liners with alumina heads compared to cobalt-chrome heads in hip replacements: a 10-year follow-up with radiostereometry in 43 hips. Acta Orthop 2013; 84(4): 360-4. doi: 10.3109/17453674.2013.810516.

44. Dahl J, Söderlund P, Nivbrant B, Nordsletten L, Röhrl SM. Less wear with aluminium-oxide heads than cobalt-chrome heads with ultra high molecular weight cemented polyethylene cups: a ten-year follow-up with radiostereometry. Int Orthop 2012; 36(3): 485-90. doi: 10.1007/s00264-011-1334-3.

45. de Ridder R, Kaptein BL, Pijls BG, Nelissen R, Kaptijn HH. Five-year migration and insert wear of uncemented tibial components with either conventional polyethylene or sequentially annealed highly crosslinked polyethylene inserts: a blinded randomized controlled trial using radiostereometric analysis. Bone Joint J 2023; 105-b(5): 518-25. doi: 10.1302/0301-620x.105b5.Bjj-2022-0986.R1.

46. de Waard S, Sierevelt IN, Jonker R, Hoornenborg D, van der Vis HM, Kerkhoffs G, et al. The migration pattern and initial stability of the Optimys short stem in total hip arthroplasty: a prospective 2-year follow-up study of 33 patients with RSA. Hip Int 2021; 31(4): 507-15. doi: 10.1177/1120700020901844.

47. deVos MJ, Verdonschot N, Luites JW, Anderson PG, Eygendaal D. Stable fixation of the IBP humeral component implanted without cement in total elbow replacement: a radiostereometric analysis study of 16 elbows at two-year follow-up. Bone Joint J 2014; 96-b(2): 229-36. doi: 10.1302/0301-620x.96b2.29050.

48. Dunbar MJ, Fong JW, Wilson DA, Hennigar AW, Francis PA, Glazebrook MA. Longitudinal migration and inducible displacement of the Mobility Total Ankle System. Acta Orthop 2012; 83(4): 394-400. doi: 10.3109/17453674.2012.712890.

49. Dunbar MJ, Laende EK, Collopy D, Richardson CG. Stable migration of peri-apatite-coated uncemented tibial components in a multicentre study. Bone Joint J 2017; 99-b(12): 1596-602. doi: 10.1302/0301-620x.99b12.Bjj-2016-1118.R2.

50. Dyreborg K, Andersen MR, Winther N, Solgaard S, Flivik G, Petersen MM. Migration of the uncemented Echo Bi-Metric and Bi-Metric THA stems: a randomized controlled RSA study involving 62 patients with 24-month follow-up. Acta Orthop 2020; 91(6): 693-8. doi: 10.1080/17453674.2020.1802682.

51. Dyreborg K, Sørensen MS, Flivik G, Solgaard S, Petersen MM. Preoperative BMD does not influence femoral stem subsidence of uncemented THA when the femoral T-score is > -2.5. Acta Orthop 2021; 92(5): 538-43. doi: 10.1080/17453674.2021.1920163.

52. Dyreborg K, Winther N, Lind T, Flivik G, Mørk Petersen M. Evaluation of different coatings of the tibial tray in uncemented total knee arthroplasty. A randomized controlled trial with 5 years follow-up with RSA and DEXA. Knee 2021; 29: 208-15. doi: 10.1016/j.knee.2021.02.002.

53. Ebert JR, Nivbrant NO, Petrov V, Yates P, Wood DJ. A 2-year prospective clinical and bone density evaluation, with a subset undergoing radiostereometric analysis, using the Absolut cemented stem. ANZ J Surg 2022; 92(4): 830-6. doi: 10.1111/ans.17519.

54. Edmondson M, Ebert J, Nivbrant O, Wood D. Prospective randomised clinical trial assessing subsidence and rotation, using radiostereometric analysis, of two modular cementless femoral stems (Global K2 and Apex). Journal of Orthopaedics 2014; 11(2): 96-102. doi: https: //dx.doi.org/10.1016/j.jor.2014.02.001.

55. Ejaz A, Laursen AC, Jakobsen T, Rasmussen S, Nielsen PT, Laursen MB. Absence of a Tourniquet Does Not Affect Fixation of Cemented TKA: A Randomized RSA Study of 70 Patients. J Arthroplasty 2015; 30(12): 2128-32. doi: 10.1016/j.arth.2015.05.058.

56. El-Sahoury JAN, Kjærgaard K, Ovesen O, Hofbauer C, Overgaard S, Ding M. Vitamin E-diffused liners show less head penetration than cross-linked polyethylene liners in total hip arthroplasty: a ten-year multi-arm randomized trial. Bone Joint J 2023; 105-b(10): 1052-9. doi: 10.1302/0301-620x.105b10.Bjj-2023-0115.R1.

57. Ensini A, Barbadoro P, Leardini A, Catani F, Giannini S. Early migration of the cemented tibial component of unicompartmental knee arthroplasty: a radiostereometry study. Knee Surg Sports Traumatol Arthrosc 2013; 21(11): 2474-9. doi: 10.1007/s00167-012-2068-5.

58. Fallahnezhad K, Callary SA, O'Rourke D, Bahl JS, Thewlis D, Solomon LB, et al. Corroboration of coupled musculoskeletal model and finite element predictions with in vivo RSA migration of an uncemented acetabular component. J Orthop Res 2024; 42(2): 373-84. doi: 10.1002/jor.25671.

59. Ferguson RJ, Broomfield JA, Malak TT, Palmer AJR, Whitwell D, Kendrick B, et al. Primary stability of a short bone-conserving femoral stem: a two-year randomized controlled trial using radiostereometric analysis. Bone Joint J 2018; 100-b(9): 1148-56. doi: 10.1302/0301-620x.100b9.Bjj-2017-1403.R1.

60. Finnilä S, Löyttyniemi E, Aro HT. Denosumab in Cementless Total Hip Arthroplasty: Multivariate Reanalysis of 3D Femoral Stem Migration and the Influence on Outliers. JBMR Plus 2022; 6(2): e10588. doi: 10.1002/jbm4.10588.

61. Finnilä S, Moritz N, Svedströ ME, Alm JJ, Aro HT. Increased migration of uncemented acetabular cups in female total hip arthroplasty patients with low systemic bone mineral density. A 2-year RSA and 8-year radiographic follow-up study of 34 patients. Acta Orthop 2016; 87(1): 48-54. doi: 10.3109/17453674.2015.1115312.

62. Flatøy B, Dahl J, Röhrl SM, Nordsletten L. Does radiopaque cement conceal periprosthetic bone loss around femoral stems? Hip Int 2020; 30(6): 731-8. doi: 10.1177/1120700019863352.

63. Flatøy B, Röhrl SM, Bøe B, Nordsletten L. No medium-term advantage of electrochemical deposition of hydroxyapatite in cementless femoral stems. 5-year RSA and DXA results from a randomized controlled trial. Acta Orthop 2016; 87(1): 42-7. doi: 10.3109/17453674.2015.1084768.

64. Flatøy B, Röhrl SM, Rydinge J, Dahl J, Diep LM, Nordsletten L. Triple taper stem design shows promising fixation and bone remodelling characteristics: radiostereometric analysis in a randomised controlled trial. Bone Joint J 2015; 97-b(6): 755-61. doi: 10.1302/0301-620x.97b6.34736.

65. Flatøy B, Rydinge J, Dahl J, Röhrl SM, Nordsletten L. Low wear, high stability - promises of success in a moderately cross-linked cup? Hip Int 2015; 25(3): 199-203. doi: 10.5301/hipint.5000230.

66. Flivik G, Kristiansson I, Ryd L. Positive effect of removal of subchondral bone plate for cemented acetabular component fixation in total hip arthroplasty: a randomised RSA study with ten-year follow-up. Bone Joint J 2015; 97-b(1): 35-44. doi: 10.1302/0301-620x.97b1.34391.

67. Floerkemeier T, Budde S, Lewinski GV, Windhagen H, HurSchler C, Schwarze M. Greater early migration of a short-stem total hip arthroplasty is not associated with an increased risk of osseointegration failure: 5th-year results from a prospective RSA study with 39 patients, a follow-up study. Acta Orthop 2020; 91(3): 266-71. doi: 10.1080/17453674.2020.1732749.

68. Fontalis A, Kayani B, Vanhegan I, Tahmassebi J, Haddad IC, Giebaly DE, et al 2-Year Radiostereometric Analysis Evaluation of a Short, Proximally Coated, Triple-Taper Blade Femoral Stem Versus a Quadrangular-Taper Stem With Reinforced Proximal Body: A Randomized Controlled Trial. J Arthroplasty 2023; 38(7s): S152-s61. doi: 10.1016/j.arth.2023.03.030.

69. Fraser AN, Bøe B, Fjalestad T, Madsen JE, Röhrl SM. Stable glenoid component of reverse total shoulder arthroplasty at 2 years as measured with model-based radiostereometric analysis (RSA). Acta Orthop 2021; 92(6): 644-50. doi: 10.1080/17453674.2021.1943932.

70. Galea VP, Rojanasopondist P, Laursen M, Muratoglu OK, Malchau H, Bragdon C. Evaluation of vitamin E-diffused highly crosslinked polyethylene wear and porous titanium-coated shell stability: a seven-year randomized control trial using radiostereometric analysis. Bone Joint J 2019; 101-b(7): 760-7. doi: 10.1302/0301-620x.101b7.Bjj-2019-0268.R1.

71. Gascoyne TC, McRae SMB, Parashin SL, Leiter JRS, Petrak MJ, Bohm ER, et al. Radiostereometric analysis of keeled versus pegged glenoid components in total shoulder arthroplasty: a randomized feasibility study. Can J Surg 2017; 60(4): 273-9. doi: 10.1503/cjs.001817.

72. Gudnason A, Adalberth G, Nilsson KG, Hailer NP. Tibial component rotation around the transverse axis measured by radiostereometry predicts aseptic loosening better than maximal total point motion. Acta Orthop 2017; 88(3): 282-7. doi: 10.1080/17453674.2017.1297001.

73. Hansen TB, Stilling M. Equally good fixation of cemented and uncemented cups in total trapeziometacarpal joint prostheses. A randomized clinical RSA study with 2-year follow-up. Acta Orthop 2013; 84(1): 98-105. doi: 10.3109/17453674.2013.765625.

74. Hasan S, Kaptein BL, Marang-van de Mheen PJ, Van Hamersveld KT, Nelissen R, Toksvig-Larsen S. Late stabilization after initial migration in patients undergoing cemented total knee arthroplasty: a 5-year followup of 2 randomized controlled trials using radiostereometric analysis. Acta Orthop 2022; 93: 271-6. doi: 10.2340/17453674.2022.1381.

75. Hasan S, Kaptein BL, Nelissen R, van Hamersveld KT, Toksvig-Larsen S, Marang-van de Mheen PJ. The Influence of Postoperative Coronal Alignment on Tibial Migration After Total Knee Arthroplasty in Preoperative Varus and Valgus Knees: A Secondary Analysis of 10 Randomized Controlled Trials Using Radiostereometric Analysis. J Bone Joint Surg Am 2021; 103(24): 2281-90. doi: 10.2106/jbjs.20.01659.

76. Hasan S, Marang-Van De Mheen PJ, Kaptein BL, Nelissen R, Toksvig-Larsen S. All-polyethylene versus metal-backed posterior stabilized total knee arthroplasty: similar 2-year results of a randomized radiostereometric analysis study. Acta Orthop 2019; 90(6): 590-5. doi: 10.1080/17453674.2019.1668602.

77. Hasan S, van Hamersveld KT, Marang-van de Mheen PJ, Kaptein BL, Nelissen R, Toksvig-Larsen S. Migration of a novel 3D-printed cementless versus a cemented total knee arthroplasty: two-year results of a randomized controlled trial using radiostereometric analysis. Bone Joint J 2020; 102-b(8): 1016-24. doi: 10.1302/0301-620x.102b8.Bjj-2020-0054.R1.

78. Haugan K, Foss OA, Husby OS, Husby VS, Svenningsen S, Winther SB. Surgical approach had minor association with femoral stem migration in total hip arthroplasty: radiostereometric analysis of 61 patients after 5-year follow-up. Acta Orthop 2023; 94: 410-5. doi: 10.2340/17453674.2023.18264.

79. Haugan K, Husby OS, Klaksvik J, Foss OA. The migration pattern of the Charnley femoral stem: a five-year follow-up RSA study in a well-functioning patient group. J Orthop Traumatol 2012; 13(3): 137-43. doi: 10.1007/s10195-012-0187-x.

80. Heesterbeek PJ, Wymenga AB, van Hellemondt GG. No Difference in Implant Micromotion Between Hybrid Fixation and Fully Cemented Revision Total Knee Arthroplasty: A Randomized Controlled Trial with Radiostereometric Analysis of Patients with Mild-to-Moderate Bone Loss. J Bone Joint Surg Am 2016; 98(16): 1359-69. doi: 10.2106/jbjs.15.00909.

81. Henricson A, Nilsson KG. Trabecular metal tibial knee component still stable at 10 years. Acta Orthop 2016; 87(5): 504-10. doi: 10.1080/17453674.2016.1205169.

82. Henricson A, Rösmark D, Nilsson KG. Trabecular metal tibia still stable at 5 years: an RSA study of 36 patients aged less than 60 years. Acta Orthop 2013; 84(4): 398-405. doi: 10.3109/17453674.2013.799418.

83. Henricson A, Wojtowicz R, Nilsson KG, Crnalic S. Uncemented or cemented femoral components work equally well in total knee arthroplasty. Knee Surg Sports Traumatol Arthrosc 2019; 27(4): 1251-8. doi: 10.1007/s00167-018-5227-5.

84. Hjorth MH, Kold S, Søballe K, Langdahl BL, Nielsen PT, Christensen PH, et al. Preparation of the Femoral Bone Cavity for Cementless Stems: Broaching vs Compaction. A Five-Year Randomized Radiostereometric Analysis and Dual Energy X-Ray Absorption Study. J Arthroplasty 2017; 32(6): 1894-901. doi: 10.1016/j.arth.2016.12.029.

85. Hjorth MH, Lorenzen ND, Søballe K, Jakobsen SS, Stilling M. Equal Primary Fixation of Resurfacing Stem, but Inferior Cup Fixation With Anterolateral vs Posterior Surgical Approach. A 2-Year Blinded Randomized Radiostereometric and Dual-energy X-Ray Absorptiometry Study of Metal-on-Metal Hip Resurfacing Arthroplasty. J Arthroplasty 2017; 32(11): 3412-20. doi: 10.1016/j.arth.2017.05.034.

86. Hjorth MH, Søballe K, Jakobsen SS, Lorenzen ND, Mechlenburg I, Stilling M. No association between serum metal ions and implant fixation in large-head metal-on-metal total hip arthroplasty. Acta Orthop 2014; 85(4): 355-62. doi: 10.3109/17453674.2014.922731.

87. Hjorth MH, Stilling M, Søballe K, Nielsen PT, Christensen PH, Kold S. Preparation of the femoral bone cavity in cementless stems: broaching versus compaction. Acta Orthop 2016; 87(6): 575-82. doi: 10.1080/17453674.2016.1244958.

88. Holm-Glad T, Røkkum M, Röhrl SM, Roness S, Godang K, Reigstad O. A randomized controlled trial comparing two modern total wrist arthroplasties : improved function with stable implants, but high complication rates in non-rheumatoid wrists at two years. Bone Joint J 2022; 104-b(10): 1132-41. doi: 10.1302/0301-620x.104b10.Bjj-2022-0201.R2.

89. Hoornenborg D, Schweden AMC, Sierevelt IN, van der Vis HM, Kerkhoffs G, Haverkamp D. The influence of hydroxyapatite coating on continuous migration of a Zweymuller-type hip stem: a double-blinded randomised RSA trial with 5-year follow-up. Hip Int 2023; 33(1): 73-80. doi: 10.1177/11207000211006782.

90. Hoornenborg D, Sierevelt IN, Spuijbroek JA, Cheung J, van der Vis HM, Beimers L, et al. Does hydroxyapatite coating enhance ingrowth and improve longevity of a Zweymuller type stem? A double-blinded randomised RSA trial. Hip Int 2018; 28(2): 115-21. doi: 10.5301/hipint.5000549.

91. Howie DW, Holubowycz OT, Callary SA, Robertson TS, Solomon LB. Highly Porous Tantalum Acetabular Components Without Ancillary Screws Have Similar Migration to Porous Titanium Acetabular Components With Screws at 2 Years: A Randomized Controlled Trial. J Arthroplasty 2020; 35(10): 2931-7. doi: 10.1016/j.arth.2020.05.049.

92. Itayem R, Arndt A, Daniel J, McMinn DJ, Lundberg A. A two-year radiostereometric follow-up of the first generation Birmingham mid head resection arthroplasty. Hip Int 2014; 24(4): 355-62. doi: 10.5301/hipint.5000136.

93. Jacobsen A, Seehaus F, Hong Y, Cao H, Schuh A, Forst R, et al. Model-based roentgen stereophotogrammetric analysis using elementary geometrical shape models: 10 years results of an uncemented acetabular cup component. BMC Musculoskelet Disord 2018; 19(1): 335. doi: 10.1186/s12891-018-2259-4.

94. Jensen CL, Petersen MM, Schrøder HM, Flivik G, Lund B. Revision total knee arthroplasty with the use of trabecular metal cones: a randomized radiostereometric analysis with 2 years of follow-up. J Arthroplasty 2012; 27(10): 1820-6.e2. doi: 10.1016/j.arth.2012.04.036.

95. Johanson PE, Antonsson M, Shareghi B, Kärrholm J. Early Subsidence Predicts Failure of a Cemented Femoral Stem With Minor Design Changes. Clin Orthop Relat Res 2016; 474(10): 2221-9. doi: 10.1007/s11999-016-4884-2.

96. Johanson PE, Digas G, Herberts P, Thanner J, Kärrholm J. Highly crosslinked polyethylene does not reduce aseptic loosening in cemented THA 10-year findings of a randomized study. Clin Orthop Relat Res 2012; 470(11): 3083-93. doi: 10.1007/s11999-012-2400-x.

97. Jørgensen PB, Daugaard H, Jakobsen SS, Lamm M, Søballe K, Stilling M. Higher early proximal migration of hemispherical cups with electrochemically applied hydroxyapatite (BoneMaster) on a porous surface compared with porous surface alone: a randomized RSA study with 53 patients. Acta Orthop 2020; 91(1): 26-32. doi: 10.1080/17453674.2019.1687860.

98. Jørgensen PB, Kaptein BL, Søballe K, Jakobsen SS, Stilling M. Five-year polyethylene cup migration and PE wear of the Anatomic Dual Mobility acetabular construct. Arch Orthop Trauma Surg 2023; 143(9): 5957-65. doi: 10.1007/s00402-023-04774-5.

99. Jørgensen PB, Lamm M, Søballe K, Stilling M. Equivalent hip stem fixation by Hi-Fatigue G and Palacos R + G bone cement: a randomized radiostereometric controlled trial of 52 patients with 2 years' follow-up. Acta Orthop 2019; 90(3): 237-42. doi: 10.1080/17453674.2019.1595390.

100. Jørgensen PB, Tabori-Jensen S, Mechlenburg I, Homilius M, Hansen TB, Stilling M. Cemented and cementless dual mobility cups show similar fixation, low polyethylene wear, and low serum cobalt-chromium in elderly patients: a randomized radiostereometry study with 6 years' follow-up. Acta Orthop 2022; 93: 906-13. doi: 10.2340/17453674.2022.5761.

101. Jun BJ, Ricchetti ET, Haladik J, Bey MJ, Patterson TE, Subhas N, et al. Validation of a 3D CT imaging method for quantifying implant migration following anatomic total shoulder arthroplasty. J Orthop Res 2022; 40(6): 1270-80. doi: 10.1002/jor.25170.

102. Kadar T, Furnes O, Aamodt A, Indrekvam K, Havelin LI, Haugan K, et al. The influence of acetabular inclination angle on the penetration of polyethylene and migration of the acetabular component: a prospective, radiostereometric study on cemented acetabular components. J Bone Joint Surg Br 2012; 94(3): 302-7. doi: 10.1302/0301-620x.94b3.27460.

103. Kaptein BL, den Hollander P, Thomassen B, Fiocco M, Nelissen R. A randomized controlled trial comparing tibial migration of the ATTUNE cemented cruciate-retaining knee prosthesis with the PFC-sigma design. Bone Joint J 2020; 102-b(9): 1158-66. doi: 10.1302/0301-620x.102b9.Bjj-2020-0096.R1.

104. Keiller T, Saari T, Sharegi B, Kärrholm J. No difference in clinical outcome but in RSA in total knee arthroplasty with the ATTUNE vs. the PFC Sigma: a randomized trial with 2-year follow-up. Acta Orthop 2023; 94: 560-9. doi: 10.2340/17453674.2023.24577.

105. Kendrick BJ, Kaptein BL, Valstar ER, Gill HS, Jackson WF, Dodd CA, et al. Cemented versus cementless Oxford unicompartmental knee arthroplasty using radiostereometric analysis: a randomised controlled trial. Bone Joint J 2015; 97-b(2): 185-91. doi: 10.1302/0301-620x.97b2.34331.

106. Kent M, Edmondson M, Ebert J, Nivbrant N, Kop A, Wood D, et al. Stem Migration and Fretting Corrosion of the Antirotation Pin in the K2/Apex Hip System. J Arthroplasty 2016; 31(3): 727-34. doi: 10.1016/j.arth.2015.10.004.

107. Kiernan S, Geijer M, Sundberg M, Flivik G. Effect of symmetrical restoration for the migration of uncemented total hip arthroplasty: a randomized RSA study with 75 patients and 5-year follow-up. J Orthop Surg Res 2020; 15(1): 225. doi: 10.1186/s13018-020-01736-0.

108. Kiernan S, Hermann KL, Wagner P, Ryd L, Flivik G. The importance of adequate stem anteversion for rotational stability in cemented total hip replacement: a radiostereometric study with ten-year follow-up. Bone Joint J 2013; 95-b(1): 23-30. doi: 10.1302/0301-620x.95b1.30055.

109. Kjærgaard K, Ding M, Jensen C, Bragdon C, Malchau H, Andreasen CM, et al. Vitamin E-doped total hip arthroplasty liners show similar head penetration to highly cross-linked polyethylene at five years: a multi-arm randomized controlled trial. Bone Joint J 2020; 102-b(10): 1303-10. doi: 10.1302/0301-620x.102b10.Bjj-2020-0138.R1.

110. Klaassen AD, Schäffer EA, Willigenburg NW, Van Beers L, Scholtes VAB, Van der Hulst VPM, et al. Comparison of early migration patterns between a ceramic and polyethylene liner in uncemented Trabecular Titanium cups: a 2-year randomized controlled trial of 52 hips using radiostereometric analysis. Acta Orthop 2022; 93: 451-8. doi: 10.2340/17453674.2022.2267.

111. Klein LJ, Puretic G, Mohaddes M, Kärrholm J. Similar clinical results and early subsidence between the Collum Femoris Preserving and the Corail stem: a randomized radiostereometric study of 77 hips with 2 years' follow-up. Acta Orthop 2019; 90(3): 202-8. doi: 10.1080/17453674.2019.1577344.

112. Klerken T, Mohaddes M, Nemes S, Kärrholm J. High early migration of the revised acetabular component is a predictor of late cup loosening: 312 cup revisions followed with radiostereometric analysis for 2-20 years. Hip Int 2015; 25(5): 471-6. doi: 10.5301/hipint.5000246.

113. Knudsen MB, Thillemann JK, Jørgensen PB, Jakobsen SS, Daugaard H, Søballe K, et al. Electrochemically applied hydroxyapatite on the cementless porous surface of Bi-Metric stems reduces early migration and has a lasting effect : an efficacy trial of a randomized five-year follow-up radiostereometric study. Bone Joint J 2022; 104-b(6): 647-56. doi: 10.1302/0301-620x.104b6.Bjj-2021-1545.R1.

114. Kok RY, Koster LA, Kaptein BL, Fiocco M, Keizer SB. A model-based radiostereometric analysis (RSA) randomized control trial evaluating the stability of the cementless Taperloc hip stem: the TapHip study. Acta Orthop 2022; 93: 212-21. doi: 10.2340/17453674.2021.1127.

115. Koppens D, Rytter S, Dalsgaard J, Sørensen OG, Hansen TB, Stilling M. The Effect of Bone Quality on Tibial Component Migration in Medial Cemented Unicompartmental Knee Arthroplasty. A Prospective Cohort Study Using Dual X-Ray Absorptiometry and Radiostereometric Analysis. J Arthroplasty 2020; 35(3): 675-82.e2. doi: 10.1016/j.arth.2019.10.027.

116. Koppens D, Rytter S, Munk S, Dalsgaard J, Sørensen OG, Hansen TB, et al. Equal tibial component fixation of a mobile-bearing and fixed-bearing medial unicompartmental knee arthroplasty: a randomized controlled RSA study with 2-year follow-up. Acta Orthop 2019; 90(6): 575-81. doi: 10.1080/17453674.2019.1639965.

117. Koppens D, Stilling M, Munk S, Dalsgaard J, Rytter S, Sørensen OG, et al. Low implant migration of the SIGMA(®) medial unicompartmental knee arthroplasty. Knee Surg Sports Traumatol Arthrosc 2018; 26(6): 1776-85. doi: 10.1007/s00167-017-4782-5.

118. Kosse NM, van Hellemondt GG, Wymenga AB, Heesterbeek PJ. Comparable Stability of Cemented vs Press-Fit Placed Stems in Revision Total Knee Arthroplasty With Mild to Moderate Bone Loss: 6.5-Year Results From a Randomized Controlled Trial With Radiostereometric Analysis. J Arthroplasty 2017; 32(1): 197-201. doi: 10.1016/j.arth.2016.06.003.

119. Koster LA, Meinardi JE, Kaptein BL, Van der Linden-Van der Zwaag E, Nelissen R. Two-year RSA migration results of symmetrical and asymmetrical tibial components in total knee arthroplasty: a randomized controlled trial. Bone Joint J 2021; 103-b(5): 855-63. doi: 10.1302/0301-620x.103b5.Bjj-2020-1575.R2.

120. Koster LA, Rassir R, Kaptein BL, Sierevelt IN, Schager M, Nelissen R, et al. A randomized controlled trial comparing two-year postoperative femoral and tibial migration of a new and an established cementless rotating platform total knee arthroplasty. Bone Joint J 2023; 105-b(2): 148-57. doi: 10.1302/0301-620x.105b2.Bjj-2022-0414.R1.

121. Kruijntjens D, Koster L, Kaptein BL, Jutten LMC, Arts JJ, Ten Broeke RHM. Early stabilization of the uncemented Symax hip stem in a 2-year RSA study. Acta Orthop 2020; 91(2): 159-64. doi: 10.1080/17453674.2019.1709956.

122. Laende EK, Astephen Wilson JL, Mills Flemming J, Valstar ER, Richardson CG, Dunbar MJ. Equivalent 2-year stabilization of uncemented tibial component migration despite higher early migration compared with cemented

fixation: an RSA study on 360 total knee arthroplasties. Acta Orthop 2019; 90(2): 172-8. doi: 10.1080/17453674.2018.1562633.

123. Laende EK, Mills Flemming J, Astephen Wilson JL, Cantoni E, Dunbar MJ. The associations of implant and patient factors with migration of the tibial component differ by sex : a radiostereometric study on more than 400 total knee arthroplasties. Bone Joint J 2022; 104-b(4): 444-51. doi: 10.1302/0301-620x.104b4.Bjj-2021-1247.R1.

124. Laende EK, Richardson CG, Dunbar MJ. A randomized controlled trial of tibial component migration with kinematic alignment using patient-specific instrumentation versus mechanical alignment using computer-assisted surgery in total knee arthroplasty. Bone Joint J 2019; 101-b(8): 929-40. doi: 10.1302/0301-620x.101b8.Bjj-2018-0755.R3.

125. Laende EK, Richardson CG, Dunbar MJ. Predictive value of short-term migration in determining long-term stable fixation in cemented and cementless total knee arthroplasties. Bone Joint J 2019; 101-b(7_Supple_C): 55-60. doi: 10.1302/0301-620x.101b7.Bjj-2018-1493.R1.

126. Laende EK, Richardson CG, Dunbar MJ. Migration and Wear of a Dual Mobility Acetabular Construct at 3 Years Measured by Radiostereometric Analysis. J Arthroplasty 2020; 35(4): 1109-16. doi: 10.1016/j.arth.2019.11.010.

127. Laende EK, Richardson CG, Meldrum AR, Dunbar MJ. Tibial Component Migration After Total Knee Arthroplasty With High-Viscosity Bone Cement. J Arthroplasty 2021; 36(6): 2000-5. doi: 10.1016/j.arth.2021.01.081.

128. Lam Tin Cheung K, Lanting BA, McCalden RW, Yuan X, MacDonald SJ, Naudie DD, et al. Inducible displacement of cemented tibial components ten years after total knee arthroplasty. Bone Joint J 2018; 100-b(2): 170-5. doi: 10.1302/0301-620x.100b2.Bjj-2017-0428.R2.

129. Lazarinis S, Mattsson P, Milbrink J, Mallmin H, Hailer NP. A prospective cohort study on the short collum femoris-preserving (CFP) stem using RSA and DXA. Primary stability but no prevention of proximal bone loss in 27 patients followed for 2 years. Acta Orthop 2013; 84(1): 32-9. doi: 10.3109/17453674.2013.765623.

130. Lazarinis S, Milbrink J, Mattsson P, Mallmin H, Hailer NP. Bone loss around a stable, partly threaded hydroxyapatite-coated cup: a prospective cohort study using RSA and DXA. Hip Int 2014; 24(2): 155-66. doi: 10.5301/hipint.5000104.

131. Ledin H, Aspenberg P, Good L. Tourniquet use in total knee replacement does not improve fixation, but appears to reduce final range of motion. Acta Orthop 2012; 83(5): 499-503. doi: 10.3109/17453674.2012.727078.

132. Ledin H, Good L, Aspenberg P. Denosumab reduces early migration in total knee replacement. Acta Orthop 2017; 88(3): 255-8. doi: 10.1080/17453674.2017.1300746.

133. Ledin H, Good L, Johansson T, Aspenberg P. No effect of teriparatide on migration in total knee replacement. Acta Orthop 2017; 88(3): 259-62. doi: 10.1080/17453674.2017.1300745.

134. Li Y, Röhrl SM, Bøe B, Nordsletten L. Comparison of two different Radiostereometric analysis (RSA) systems with markerless elementary geometrical shape modeling for the measurement of stem migration. Clin Biomech (Bristol, Avon) 2014; 29(8): 950-5. doi: 10.1016/j.clinbiomech.2014.06.007.

135. Lindalen E, Dahl J, Nordsletten L, Snorrason F, Høvik Ø, Röhrl S. Reverse hybrid and cemented hip replacement compared using radiostereometry and dual-energy X-ray absorptiometry: 43 hips followed for 2 years in a prospective trial. Acta Orthop 2012; 83(6): 592-8. doi: 10.3109/17453674.2012.742393.

136. Lindalen E, Nordsletten L, Röhrl SM. Segment choice and cup stability influence wear measurements using radiostereometric analysis: a radiostereometric study comparing wear measured by markers in the polyethylene with markers in the periacetabular bone. Clin Biomech (Bristol, Avon) 2012; 27(5): 511-4. doi: 10.1016/j.clinbiomech.2011.11.009.

137. Linde KN, Madsen F, Puhakka KB, Langdahl BL, Søballe K, Krog-Mikkelsen I, et al. Preoperative Systemic Bone Quality Does Not Affect Tibial Component Migration in Knee Arthroplasty: A 2-Year Radiostereometric Analysis of a Hundred Consecutive Patients. J Arthroplasty 2019; 34(10): 2351-9. doi: 10.1016/j.arth.2019.05.019.

138. Linde KN, Rytter S, Søballe K, Madsen F, Langdahl B, Stilling M. Component migration, bone mineral density changes, and bone turnover markers in cementless and cemented total knee arthroplasty: a prospective randomized RSA study in 53 patients with 2-year follow-up. Knee Surg Sports Traumatol Arthrosc 2022; 30(9): 3100-13. doi: 10.1007/s00167-022-06860-4.

139. Lorenzen ND, Stilling M, Jakobsen SS, Gustafson K, Søballe K, Baad-Hansen T. Marker-based or model-based RSA for evaluation of hip resurfacing arthroplasty? A clinical validation and 5-year follow-up. Arch Orthop Trauma Surg 2013; 133(11): 1613-21. doi: 10.1007/s00402-013-1850-2.

140. Mahmoud AN, Kesteris U, Flivik G. Stable migration pattern of an ultra-short anatomical uncemented hip stem: a prospective study with 2 years radiostereometric analysis follow-up. Hip Int 2017; 27(3): 259-66. doi: 10.5301/hipint.5000458.

141. Matejcic A, Vidovic D, Nebergall A, Greene M, Bresina S, Tepic S, et al. New cementless fixation in hip arthroplasty: a radiostereometric analysis. Hip Int 2015; 25(5): 477-83. doi: 10.5301/hipint.5000254.

142. McCalden RW, Korczak A, Somerville L, Yuan X, Naudie DD. A randomised trial comparing a short and a standard-length metaphyseal engaging cementless femoral stem using radiostereometric analysis. Bone Joint J 2015; 97-b(5): 595-602. doi: 10.1302/0301-620x.97b5.34994.

143. Mechlenburg I, Klebe TM, Døssing KV, Amstrup A, Søballe K, Stilling M. Evaluation of periprosthetic bone mineral density and postoperative migration of humeral head resurfacing implants: two-year results of a randomized controlled clinical trial. J Shoulder Elbow Surg 2014; 23(10): 1427-36. doi: 10.1016/j.jse.2014.05.012.

144. Meinardi JE, Valstar ER, Van Der Voort P, Kaptein BL, Fiocco M, Nelissen RG. Palacos compared to Palamed bone cement in total hip replacement: a randomized controlled trial. Acta Orthop 2016; 87(5): 473-8. doi: 10.1080/17453674.2016.1199146.

145. Mills K, Wymenga AB, Bénard MR, Kaptein BL, Defoort KC, van Hellemondt GG, et al. Fluoroscopic and radiostereometric analysis of a bicruciate-retaining versus a posterior cruciate-retaining total knee arthroplasty: a randomized controlled trial. Bone Joint J 2023; 105-b(1): 35-46. doi: 10.1302/0301-620x.105b1.Bjj-2022-0465.R2.

146. Mills K, Wymenga AB, van Hellemondt GG, Heesterbeek PJC. No difference in long-term micromotion between fully cemented and hybrid fixation in revision total knee arthroplasty: a randomized controlled trial. Bone Joint J 2022; 104-b(7): 875-83. doi: 10.1302/0301-620x.104b7.Bjj-2021-1600.R1.

147. Minten MJ, Heesterbeek PJ, Spruit M. No effect of additional screw fixation of a cementless, all-polyethylene press-fit socket on migration, wear, and clinical outcome. Acta Orthop 2016; 87(4): 363-7. doi: 10.1080/17453674.2016.1190244.

148. Mohaddes M, Herberts P, Malchau H, Johanson PE, Kärrholm J. High proximal migration in cemented acetabular revisions operated with bone impaction grafting; 47 revision cups followed with RSA for 17 years. Hip Int 2017; 27(3): 251-8. doi: 10.5301/hipint.5000452.

149. Mohaddes M, Shareghi B, Kärrholm J. Promising early results for trabecular metal acetabular components used at revision total hip arthroplasty: 42 acetabular revisions followed with radiostereometry in a prospective randomised trial. Bone Joint J 2017; 99-b(7): 880-6. doi: 10.1302/0301-620x.99b7.Bjj-2016-1241.R1.

150. Molt M, Harsten A, Toksvig-Larsen S. The effect of tourniquet use on fixation quality in cemented total knee arthroplasty a prospective randomized clinical controlled RSA trial. Knee 2014; 21(2): 396-401. doi: 10.1016/j.knee.2013.10.008.

151. Molt M, Ljung P, Toksvig-Larsen S. Does a new knee design perform as well as the design it replaces? Bone Joint Res 2012; 1(12): 315-23. doi: 10.1302/2046-3758.112.2000064.

152. Molt M, Ryd L, Toksvig-Larsen S. A randomized RSA study concentrating especially on continuous migration. Acta Orthop 2016; 87(3): 262-7. doi: 10.3109/17453674.2016.1166876.

153. Molt M, Toksvig-Larsen S. Similar early migration when comparing CR and PS in Triathlon™ TKA: A prospective randomised RSA trial. Knee 2014; 21(5): 949-54. doi: 10.1016/j.knee.2014.05.012.

154. Molt M, Toksvig-Larsen S 2-year follow-up report on micromotion of a short tibia stem. A prospective, randomized RSA study of 59 patients. Acta Orthop 2015; 86(5): 594-8. doi: 10.3109/17453674.2015.1033303.

155. Mosegaard SB, Odgaard A, Madsen F, Rømer L, Kristensen PW, Vind TD, et al. Comparison of cementless twin-peg, cemented twin-peg and cemented single-peg femoral component migration after medial unicompartmental knee replacement: a 5-year randomized RSA study. Arch Orthop Trauma Surg 2023; 143(12): 7169-83. doi: 10.1007/s00402-023-04991-y.

156. Munir S, Suzuki L, Dixon M. Migration Characteristics of a Proximally Coated Collarless Femoral Stem: A Prospective 2-Year Radiostereometric Analysis Study. Arthroplast Today 2023; 22: 101157. doi: 10.1016/j.artd.2023.101157.

157. Munzinger U, Guggi T, Kaptein B, Persoon M, Valstar E, Doets HC. A titanium plasma-sprayed cup with and without hydroxyapatite-coating: a randomised radiostereometric study of stability and osseointegration. Hip Int 2013; 23(1): 33-9. doi: 10.5301/hip.2013.10598.

158. Murray DW, Gulati A, Gill HS. Ten-year RSA-measured migration of the Exeter femoral stem. Bone Joint J 2013; 95-b(5): 605-8. doi: 10.1302/0301-620x.95b5.31330.

159. Naudie DD, Somerville L, Korczak A, Yuan X, McCalden RW, Holdsworth D, et al. A randomized trial comparing acetabular component fixation of two porous ingrowth surfaces using RSA. J Arthroplasty 2013; 28(8 Suppl): 48-52. doi: 10.1016/j.arth.2013.06.041.

160. Nazari-Farsani S, Vuopio M, Löyttyniemi E, Aro HT. Contributing factors to the initial femoral stem migration in cementless total hip arthroplasty of postmenopausal women. J Biomech 2021; 117: 110262. doi: 10.1016/j.jbiomech.2021.110262.

161. Nazari-Farsani S, Vuopio ME, Aro HT. Bone Mineral Density and Cortical-Bone Thickness of the Distal Radius Predict Femoral Stem Subsidence in Postmenopausal Women. J Arthroplasty 2020; 35(7): 1877-84.e1. doi: 10.1016/j.arth.2020.02.062.

162. Nebergall A, Bragdon C, Antonellis A, Kärrholm J, Brånemark R, Malchau H. Stable fixation of an osseointegated implant system for above-the-knee amputees: titel RSA and radiographic evaluation of migration and bone remodeling in 55 cases. Acta Orthop 2012; 83(2): 121-8. doi: 10.3109/17453674.2012.678799.

163. Niesen AE, Garverick AL, Howell SM, Hull ML. Low tibial baseplate migration 1 year after unrestricted kinematically aligned total knee arthroplasty using a medial conforming implant design. Knee Surg Sports Traumatol Arthrosc 2023; 31(4): 1433-42. doi: 10.1007/s00167-022-07171-4.

164. Nieuwenhuijse MJ, Valstar ER, Kaptein BL, Nelissen RG. Good diagnostic performance of early migration as a predictor of late aseptic loosening of acetabular cups: results from ten years of follow-up with Roentgen stereophotogrammetric analysis (RSA). J Bone Joint Surg Am 2012; 94(10): 874-80. doi: 10.2106/jbjs.K.00305.

165. Nieuwenhuijse MJ, Valstar ER, Kaptein BL, Nelissen RG. The Exeter femoral stem continues to migrate during its first decade after implantation: 10-12 years of follow-up with radiostereometric analysis (RSA). Acta Orthop 2012; 83(2): 129-34. doi: 10.3109/17453674.2012.672093.

166. Nieuwenhuijse MJ, Valstar ER, Nelissen RG. 5-year clinical and radiostereometric analysis (RSA) follow-up of 39 CUT femoral neck total hip prostheses in young osteoarthritis patients. Acta Orthop 2012; 83(4): 334-41. doi: 10.3109/17453674.2012.702392.

167. Nieuwenhuijse MJ, van der Voort P, Kaptein BL, van der Linden-van der Zwaag HM, Valstar ER, Nelissen RG. Fixation of high-flexion total knee prostheses: five-year follow-up results of a four-arm randomized controlled clinical and roentgen stereophotogrammetric analysis study. J Bone Joint Surg Am 2013; 95(19): e1411-11. doi: 10.2106/jbjs.L.01523.

168. Nieuwenhuijse MJ, Vehmeijer SBW, Mathijsen NMC, Keizer SB. Fixation of the short global tissue-sparing hip stem. Bone Joint J 2020; 102-b(6): 699-708. doi: 10.1302/0301-620x.102b6.Bjj-2019-1026.R2.

169. Nilsson KG, Theodoulou A, Mercer G, Quinn SJ, Krishnan J. Mid-term migration of a cementless, porous acetabular cup: A 5 year Radiostereometric analysis. J Orthop 2017; 14(4): 454-60. doi: 10.1016/j.jor.2017.07.004.

170. Nivbrant NO, Khan RJK, Fick DP, Haebich S, Smith E. Cementless Versus Cemented Tibial Fixation in Posterior Stabilized Total Knee Replacement: A Randomized Trial. J Bone Joint Surg Am 2020; 102(12): 1075-82. doi: 10.2106/jbjs.19.01010.

171. Nuttall D, Birch A, Haines JF, Trail IA. Radiostereographic analysis of a shoulder surface replacement: does hydroxyapatite have a place? Bone Joint J 2014; 96-b(8): 1077-81. doi: 10.1302/0301-620x.96b8.30534.

172. Nuttall D, Birch A, Haines JF, Watts AC, Trail IA. Early migration of a partially cemented fluted glenoid component inserted using a cannulated preparation system. Bone Joint J 2017; 99-b(5): 674-9. doi: 10.1302/0301-620x.99b5.Bjj-2016-0745.R1.

173. Nuttall D, Haines JF, Trail IA. The early migration of a partially cemented fluted pegged glenoid component using radiostereometric analysis. J Shoulder Elbow Surg 2012; 21(9): 1191-6. doi: 10.1016/j.jse.2011.07.028.

174. Nysted M, Foss OA, Klaksvik J, Benum P, Haugan K, Husby OS, et al. Small and similar amounts of micromotion in an anatomical stem and a customized cementless femoral stem in regular-shaped femurs. A 5-year follow-up randomized RSA study. Acta Orthop 2014; 85(2): 152-8. doi: 10.3109/17453674.2014.899846.

175. Nyström A, Kiritopoulos D, Mallmin H, Lazarinis S. Continuous periprosthetic bone loss but preserved stability for a collum femoris-preserving stem: follow-up of a prospective cohort study of 21 patients with dualenergy X-ray absorptiometry and radiostereometric analysis with minimum 8 years of follow-up. Acta Orthop 2022; 93: 206-11. doi: 10.2340/17453674.2021.1080.

176. Øhrn FD, Lian Ø B, Tsukanaka M, Röhrl SM. Early migration of a medially stabilized total knee arthroplasty : a radiostereometric analysis study up to two years. Bone Jt Open 2021; 2(9): 737-44. doi: 10.1302/2633-1462.29.Bjo-2021-0115.R1.

177. Øhrn FD, Van Leeuwen J, Tsukanaka M, Röhrl SM. A 2-year RSA study of the Vanguard CR total knee system: A randomized controlled trial comparing patient-specific positioning guides with conventional technique. Acta Orthop 2018; 89(4): 418-24. doi: 10.1080/17453674.2018.1470866.

178. Okowinski M, Hjorth MH, Mosegaard SB, Jürgens-Lahnstein JH, Storgaard Jakobsen S, Hedevang Christensen P, et al. Ten-year comparison of two different techniques for femoral bone cavity preparation-broaching versus compaction in patients with cementless total hip arthroplasty : a randomized radiostereometric study of 30 total hip arthroplasties in 15 patients operated bilaterally. Bone Jt Open 2021; 2(12): 1035-42. doi: 10.1302/2633-1462.212.Bjo-2021-0152.R1.

179. Olerud F, Olsson C, Flivik G. Comparison of Refobacin bone cement and palacos with gentamicin in total hip arthroplasty: an RSA study with two years follow-up. Hip Int 2014; 24(1): 56-62. doi: 10.5301/hipint.5000088.

180. Otten V, Wästerlund D, Lindbjörn J, Mertens C, Mukka S, Crnalic S, et al. Evaluation of a new cemented highly cross-linked all-polyethylene cup: a prospective and randomised study assessing wear and fixation characteristics using radiostereometric analysis. Hip Int 2022; 32(6): 779-86. doi: 10.1177/1120700021989991.

181. Otten VT, Crnalic S, Röhrl SM, Nivbrant B, Nilsson KG. Stability of Uncemented Cups - Long-Term Effect of Screws, Pegs and HA Coating: A 14-Year RSA Follow-Up of Total Hip Arthroplasty. J Arthroplasty 2016; 31(1): 156-61. doi: 10.1016/j.arth.2015.07.012.

182. Pakvis D, Luites J, van Hellemondt G, Spruit M. A cementless, elastic press-fit socket with and without screws. Acta Orthop 2012; 83(5): 481-7. doi: 10.3109/17453674.2012.720116.

183. Pasma JH, Hesseling B, De Esch N, Verburg H, Niesten DD, Mathijssen NMC. Early migration in unicompartmental knee arthroplasty: a radiostereometric study of 26 patients with 24 months of follow-up. Acta Orthop 2022; 93: 914-21. doi: 10.2340/17453674.2022.5672.

184. Penny JO, Ding M, Varmarken JE, Ovesen O, Overgaard S. Early micromovement of the Articular Surface Replacement (ASR) femoral component: two-year radiostereometry results. J Bone Joint Surg Br 2012; 94(10): 1344-50. doi: 10.1302/0301-620x.94b10.29030.

185. Perelgut ME, Polus JS, Lanting BA, Teeter MG. The effect of femoral stem collar on implant migration and clinical outcomes following direct anterior approach total hip arthroplasty. Bone Joint J 2020; 102-b(12): 1654-61. doi: 10.1302/0301-620x.102b12.Bjj-2019-1428.R1.

186. Petursson G, Fenstad AM, Gøthesen Ø, Haugan K, Dyrhovden GS, Hallan G, et al. Similar migration in computer-assisted and conventional total knee arthroplasty. Acta Orthop 2017; 88(2): 166-72. doi: 10.1080/17453674.2016.1267835.

187. Pijls BG, Valstar ER, Kaptein BL, Fiocco M, Nelissen RG. The beneficial effect of hydroxyapatite lasts: a randomized radiostereometric trial comparing hydroxyapatite-coated, uncoated, and cemented tibial components for up to 16 years. Acta Orthop 2012; 83(2): 135-41. doi: 10.3109/17453674.2012.665330.

188. Pijls BG, Valstar ER, Kaptein BL, Nelissen RG. Differences in long-term fixation between mobile-bearing and fixed-bearing knee prostheses at ten to 12 years' follow-up: a single-blinded randomised controlled radiostereometric trial. J Bone Joint Surg Br 2012; 94(10): 1366-71. doi: 10.1302/0301-620x.94b10.28858.

189. Polus JS, Perelgut ME, Vasarhelyi EM, Teeter MG, Lanting BA. Femoral stem migration after direct lateral and direct anterior total hip arthroplasty: a prospective cohort study. Can J Surg 2022; 65(4): E487-e95. doi: 10.1503/cjs.013221.

190. Polus JS, Vasarhelyi EM, Lanting BA, Teeter MG. Acetabular cup fixation with and without screws following primary total hip arthroplasty: migration evaluated by radiostereometric analysis. Hip Int 2024; 34(1): 42-8. doi: 10.1177/11207000231164711.

191. Ramasamy B, Abrahams JM, Clothier RJ, Solomon LB, Callary SA. RSA Measurements of Implant Instability in a Paprosky III Pelvic Defect with Discontinuity: A Case Report. JBJS Case Connect 2022; 12(4). doi: e22.0029610.2106/jbjs.Cc.22.00296.

192. Reiner T, Sonntag R, Kretzer JP, Clarius M, Jakubowitz E, Weiss S, et al. The Migration Pattern of a Cementless Hydroxyapatite-Coated Titanium Stem under Immediate Full Weight-Bearing-A Randomized Controlled Trial Using Model-Based RSA. J Clin Med 2020; 9(7). doi: 10.3390/jcm9072077.

193. Richardson CG, Laende EK, Gross M, Dunbar MJ. Prospective clinical study using radiostereometric analysis (RSA) to evaluate fixation of a modular cemented polished femoral stem. Hip Int 2021; 31(2): 191-5. doi: 10.1177/1120700019881429.

194. Rilby K, Mohaddes M, Kärrholm J. Similar results after five years with the use of the Fitmore or the CLS femoral components. Bone Jt Open 2023; 4(5): 306-14. doi: 10.1302/2633-1462.45.Bjo-2023-0007.R1.

195. Rilby K, Mohaddes M, Nauclér E, Kärrholm J. Similar outcome with a new anteverted or a straight standard stem: a randomized study of 72 total hip arthroplasties evaluated with clinical variables, radiostereometry, and DXA up to 2 years. Acta Orthop 2022; 93: 59-67. doi: 10.1080/17453674.2021.1993606.

196. Rilby K, Nauclér E, Mohaddes M, Kärrholm J. No difference in outcome or migration but greater loss of bone mineral density with the Collum Femoris Preserving stem compared with the Corail stem: a randomized controlled trial with five-year follow-up. Bone Joint J 2022; 104-b(5): 581-8. doi: 10.1302/0301-620x.104b5.Bjj-2021-1539.R1.

197. Röhrl SM, Nivbrant B, Nilsson KG. No adverse effects of submelt-annealed highly crosslinked polyethylene in cemented cups: an RSA study of 8 patients 10 yaers after surgery. Acta Orthop 2012; 83(2): 148-52. doi: 10.3109/17453674.2011.652889.

198. Rutherford M, Khan RJK, Fick DP, Haebich S, Nivbrant O, Kozak T. Randomised clinical trial assessing migration of uncemented primary total hip replacement stems, with and without autologous impaction bone grafting. Int Orthop 2019; 43(12): 2715-23. doi: 10.1007/s00264-019-04290-5.

199. Saari TM, Digas G, Kärrholm JN. Risedronate does not enhance fixation or BMD in revision cups: randomised study with three years follow-up. Hip Int 2014; 24(1): 49-55. doi: 10.5301/hipint.5000081.

200. Salemyr M, Muren O, Ahl T, Bodén H, Eisler T, Stark A, et al. Lower periprosthetic bone loss and good fixation of an ultra-short stem compared to a conventional stem in uncemented total hip arthroplasty. Acta Orthop 2015; 86(6): 659-66. doi: 10.3109/17453674.2015.1067087.

201. Salemyr M, Muren O, Eisler T, Bodén H, Chammout G, Stark A, et al. Porous titanium construct cup compared to porous coated titanium cup in total hip arthroplasty. A randomised controlled trial. Int Orthop 2015; 39(5): 823-32. doi: 10.1007/s00264-014-2571-z.

202. Sandberg O, Tholén S, Carlsson S, Wretenberg P. The anatomical SP-CL stem demonstrates a non-progressing migration pattern in the first year: a low dose CT-based migration study in 20 patients. Acta Orthop 2020; 91(6): 654-9. doi: 10.1080/17453674.2020.1832294.

203. Schewelov T, Ahlborg H, Sanzén L, Besjakov J, Carlsson A. Fixation of the fully hydroxyapatite-coated Corail stem implanted due to femoral neck fracture: 38 patients followed for 2 years with RSA and DEXA. Acta Orthop 2012; 83(2): 153-8. doi: 10.3109/17453674.2011.641107.

204. Schilcher J, Ivarsson I, Perlbach R, Palm L. No Difference in Periprosthetic Bone Loss and Fixation Between a Standard-Length Stem and a Shorter Version in Cementless Total Hip Arthroplasty. A Randomized Controlled Trial. J Arthroplasty 2017; 32(4): 1220-6. doi: 10.1016/j.arth.2016.11.015.

205. Schilcher J, Palm L, Ivarsson I, Aspenberg P. Local bisphosphonate reduces migration and formation of radiolucent lines adjacent to cemented acetabular components. Bone Joint J 2017; 99-b(3): 317-24. doi: 10.1302/0301-620x.99b3.Bjj-2016-0531.R1.

206. Schoeman MA, Pijls BG, Oostlander AE, Keurentjes JC, Valstar ER, Nelissen RG, et al. Innate immune response and implant loosening: Interferon gamma is inversely associated with early migration of total knee prostheses. J Orthop Res 2016; 34(1): 121-6. doi: 10.1002/jor.22988.

207. Schotanus MGM, Pilot P, Kaptein BL, Draijer WF, Tilman PBJ, Vos R, et al. No difference in terms of radiostereometric analysis between fixed- and mobile-bearing total knee arthroplasty: a randomized, single-blind, controlled trial. Knee Surg Sports Traumatol Arthrosc 2017; 25(9): 2978-85. doi: 10.1007/s00167-016-4138-6.

208. Schwarze M, Budde S, von Lewinski G, Windhagen H, Keller MC, Seehaus F, et al. No effect of conventional vs. minimally invasive surgical approach on clinical outcome and migration of a short stem total hip prosthesis at 2-year follow-up: A randomized controlled study. Clin Biomech (Bristol, Avon) 2018; 51: 105-12. doi: 10.1016/j.clinbiomech.2017.12.004.

209. Sesselmann S, Hong Y, Schlemmer F, Hussnaetter I, Mueller LA, Forst R, et al. Radiostereometric migration measurement of an uncemented Cerafit® femoral stem: 26 patients followed for 10 years. Biomed Tech (Berl) 2018; 63(6): 657-63. doi: 10.1515/bmt-2016-0251.

210. Sesselmann S, Hong Y, Schlemmer F, Wiendieck K, Söder S, Hussnaetter I, et al. Migration measurement of the cemented Lubinus SP II hip stem - a 10-year follow-up using radiostereometric analysis. Biomed Tech (Berl) 2017; 62(3): 271-8. doi: 10.1515/bmt-2015-0172.

211. Sevaldsen K, Schnell Husby O, Lian Ø B, Farran KM, Schnell Husby V. Is the French Paradox cementing philosophy superior to the standard cementing? A randomized controlled radiostereometric trial and comparative analysis. Bone Joint J 2022; 104-b(1): 19-26. doi: 10.1302/0301-620x.104b1.Bjj-2021-0325.R2.

212. Shareghi B, Johanson PE, Kärrholm J. Femoral Head Penetration of Vitamin E-Infused Highly Cross-Linked Polyethylene Liners: A Randomized Radiostereometric Study of Seventy Hips Followed for Two Years. J Bone Joint Surg Am 2015; 97(16): 1366-71. doi: 10.2106/jbjs.N.00595.

213. Shareghi B, Johanson PE, Kärrholm J. Wear of Vitamin E-Infused Highly Cross-Linked Polyethylene at Five Years. J Bone Joint Surg Am 2017; 99(17): 1447-52. doi: 10.2106/jbjs.16.00691.

214. Shareghi B, Johanson PE, Kärrholm J. Clinical evaluation of model-based radiostereometric analysis to measure femoral head penetration and cup migration in four different cup designs. J Orthop Res 2017; 35(4): 760-7. doi: 10.1002/jor.23177.

215. Sillesen NH, Greene ME, Nebergall AK, Nielsen PT, Laursen MB, Troelsen A, et al. Three Year RSA Evaluation of Vitamin E Diffused Highly Cross-linked Polyethylene Liners and Cup Stability. J Arthroplasty 2015; 30(7): 1260-4. doi: 10.1016/j.arth.2015.02.018.

216. Sköldenberg OG, Rysinska AD, Chammout G, Salemyr M, Mukka SS, Bodén H, et al. A randomized double-blind noninferiority trial, evaluating migration of a cemented vitamin E-stabilized highly crosslinked component compared with a standard polyethylene component in reverse hybrid total hip arthroplasty. Bone Joint J 2019; 101-b(10): 1192-8. doi: 10.1302/0301-620x.101b10.Bjj-2019-0456.R2.

217. Sköldenberg OG, Sjöö H, Kelly-Pettersson P, Bodén H, Eisler T, Stark A, et al. Good stability but high periprosthetic bone mineral loss and late-occurring periprosthetic fractures with use of uncemented tapered femoral stems in patients with a femoral neck fracture. Acta Orthop 2014; 85(4): 396-402. doi: 10.3109/17453674.2014.931195.

218. Söderlund P, Dahl J, Röhrl S, Nivbrant B, Nilsson KG. 10-year results of a new low-monomer cement: follow-up of a randomized RSA study. Acta Orthop 2012; 83(6): 604-8. doi: 10.3109/17453674.2012.742392.

219. Solomon LB, Abrahams JM, Callary SA, Howie DW. The Stability of the Porous Tantalum Components Used in Revision THA to Treat Severe Acetabular Defects: A Radiostereometric Analysis Study. J Bone Joint Surg Am 2018; 100(22): 1926-33. doi: 10.2106/jbjs.18.00127.

220. Solomon LB, Studer P, Abrahams JM, Callary SA, Moran CR, Stamenkov RB, et al. Does cup-cage reconstruction with oversized cups provide initial stability in THA for osteoporotic acetabular fractures? Clin Orthop Relat Res 2015; 473(12): 3811-9. doi: 10.1007/s11999-015-4460-1.

221. Sporer S, MacLean L, Burger A, Moric M. Evaluation of a 3D-printed total knee arthroplasty using radiostereometric analysis: assessment of highly porous biological fixation of the tibial baseplate and metal-backed patellar component. Bone Joint J 2019; 101-b(7_Supple_C): 40-7. doi: 10.1302/0301-620x.101b7.Bjj-2018-1466.R1.

222. Stålman A, Sköldenberg O, Martinez-Carranza N, Roberts D, Högström M, Ryd L. No implant migration and good subjective outcome of a novel customized femoral resurfacing metal implant for focal chondral lesions. Knee Surg Sports Traumatol Arthrosc 2018; 26(7): 2196-204. doi: 10.1007/s00167-017-4805-2.

223. Steiner DK, Drivsholm NS, Buchardt STE, Laursen M. The influence of migration of the exeter V40 stem on patient reported outcome measures: a 2-year follow-up of 112 total hip arthroplasties using radiostereometric analysis. Eur J Orthop Surg Traumatol 2022; 32(1): 167-74. doi: 10.1007/s00590-021-02937-x.

224. Stilling M, Mechlenburg I, Amstrup A, Soballe K, Klebe T. Precision of novel radiological methods in relation to resurfacing humeral head implants: assessment by radiostereometric analysis, DXA, and geometrical analysis. Arch Orthop Trauma Surg 2012; 132(11): 1521-30. doi: 10.1007/s00402-012-1580-x.

225. Stilling M, Mechlenburg I, Jepsen CF, Rømer L, Rahbek O, Søballe K, et al. Superior fixation and less periprosthetic stress-shielding of tibial components with a finned stem versus an I-beam block stem: a randomized RSA and DXA study with minimum 5 years' follow-up. Acta Orthop 2019; 90(2): 165-71. doi: 10.1080/17453674.2019.1566510.

226. Szerlip B, Muh S, Streit JJ, Gobezie R. Humeral Fixation in Shoulder Arthroplasty: Does Stem Geometry Matter? Seminars in Arthroplasty JSES 2012; 23(2): 103-5. doi: https: //dx.doi.org/10.1053/j.sart.2012.03.008.

227. Tabori-Jensen S, Mosegaard SB, Hansen TB, Stilling M. Inferior stabilization of cementless compared with cemented dual-mobility cups in elderly osteoarthrosis patients: a randomized controlled radiostereometry study on 60 patients with 2 years' follow-up. Acta Orthop 2020; 91(3): 246-53. doi: 10.1080/17453674.2020.1720978.

228. Teeter MG, Broberg JS, Howard JL, Lanting BA. Axial and Sagittal Rotation of Cementless Tibial Baseplates Occurs in Bone Under Joint Loading. J Arthroplasty 2023; 38(6): 1166-71. doi: 10.1016/j.arth.2023.03.002.

229. Teeter MG, Marsh JD, Howard JL, Yuan X, Vasarhelyi EM, McCalden RW, et al. A randomized controlled trial investigating the value of patient-specific instrumentation for total knee arthroplasty in the Canadian healthcare system. Bone Joint J 2019; 101-b(5): 565-72. doi: 10.1302/0301-620x.101b5.Bjj-2018-1323.R1.

230. Teeter MG, McCalden RW, Yuan X, MacDonald SJ, Naudie DD. Predictive accuracy of RSA migration thresholds for cemented total hip arthroplasty stem designs. Hip Int 2018; 28(4): 363-8. doi: 10.1177/1120700018762179.

231. Teeter MG, Naudie DD, McCalden RW, Yuan X, Holdsworth DW, MacDonald SJ, et al. Varus tibial alignment is associated with greater tibial baseplate migration at 10 years following total knee arthroplasty. Knee Surg Sports Traumatol Arthrosc 2018; 26(6): 1610-7. doi: 10.1007/s00167-017-4765-6.

232. Teeter MG, Perry K, Yuan X, Howard JL, Lanting BA. The Effects of Resection Technique on Implant Migration in Single Radius Posterior-Stabilized Total Knee Replacement. J Knee Surg 2020; 33(1): 78-83. doi: 10.1055/s-0038-1676462.

233. Teeter MG, Perry KI, Yuan X, Howard JL, Lanting BA. Contact Kinematics Correlates to Tibial Component Migration Following Single Radius Posterior Stabilized Knee Replacement. J Arthroplasty 2018; 33(3): 740-5. doi: 10.1016/j.arth.2017.09.064.

234. Teeter MG, Thoren J, Yuan X, McCalden RW, MacDonald SJ, Lanting BA, et al. Migration of a cemented fixed-bearing, polished titanium tibial baseplate (Genesis II) at ten years : a radiostereometric analysis. Bone Joint J 2016; 98-b(5): 616-21. doi: 10.1302/0301-620x.98b5.36865.

235. Ten Brinke B, Hesseling B, Eygendaal D, Hoelen MA, Mathijssen NMC. Early fixation of the humeral component in stemless total shoulder arthroplasty : a radiostereometric and clinical study with 24-month follow-up. Bone Joint J 2022; 104-b(1): 76-82. doi: 10.1302/0301-620x.104b1.Bjj-2021-0945.R1.

236. Ten Brinke B, Mathijssen NM, Blom I, Deijkers RL, Ooms EM, Kraan GA. Model-based roentgen stereophotogrammetric analysis of the surface replacement trapeziometacarpal total joint arthroplasty. J Hand Surg Eur Vol 2016; 41(9): 925-9. doi: 10.1177/1753193416629070.

237. Ten Brinke B, Mathijssen NMC, Blom IF, Koster LA, Kraan GA. A radiostereometric and clinical long-term follow-up study of the surface replacement trapeziometacarpal joint prosthesis. BMC Musculoskelet Disord 2021; 22(1): 148. doi: 10.1186/s12891-021-03957-8.

238. Thien TM, Thanner J, Kärrholm J. Fixation and bone remodeling around a low-modulus stem seven-year follow-up of a randomized study with use of radiostereometry and dual-energy x-ray absorptiometer. J Arthroplasty 2012; 27(1): 134-42.e1. doi: 10.1016/j.arth.2011.03.029.

239. Thoen PS, Nordsletten L, Pripp AH, Röhrl SM. Results of a randomized controlled trial with five-year radiostereometric analysis results of vitamin E-infused highly crosslinked versus moderately crosslinked polyethylene in reverse total hip arthroplasty. Bone Joint J 2020; 102-b(12): 1646-53. doi: 10.1302/0301-620x.102b12.Bjj-2020-0721.R1.

240. Tjørnild M, Søballe K, Hansen PM, Holm C, Stilling M. Mobile- vs. fixed-bearing total knee replacement. Acta Orthop 2015; 86(2): 208-14. doi: 10.3109/17453674.2014.968476.

241. Torle J, Thillemann JK, Petersen ET, Madsen F, Søballe K, Stilling M. Less polyethylene wear in monobloc compared to modular ultra-high-molecular-weight-polyethylene inlays in hybrid total knee arthroplasty: A 5-year randomized radiostereometry study. Knee 2021; 29: 486-99. doi: 10.1016/j.knee.2021.02.033.

242. Tøttrup M, Thillemann JK, Thillemann TM, Mechlenburg I, Klebe T, Søballe K, et al. Early offset-increasing migration predicts later revision for humeral head resurfacing implants. A randomized controlled radiostereometry trial with 10-year clinical follow-up. J Orthop Res 2022; 40(11): 2688-97. doi: 10.1002/jor.25298.

243. Troelsen A, Ingelsrud LH, Thomsen MG, Muharemovic O, Otte KS, Husted H. Are There Differences in Micromotion on Radiostereometric Analysis Between Bicruciate and Cruciate-retaining Designs in TKA? A Randomized Controlled Trial. Clin Orthop Relat Res 2020; 478(9): 2045-53. doi: 10.1097/corr.0000000000001077.

244. Tschunko F, Wagner B, Hong Y, Söder S, Wölfel R, Müller LA, et al. Radiostereometric migration analysis of the Cerafit femoral stem: 28 patients followed for 2 years. Biomed Tech (Berl) 2016; 61(3): 291-8. doi: 10.1515/bmt-2015-0004.

245. Tsikandylakis G, Mortensen KRL, Gromov K, Troelsen A, Malchau H, Mohaddes M. The Use of Porous Titanium Coating and the Largest Possible Head Do Not Affect Early Cup Fixation: A 2-Year Report from a Randomized Controlled Trial. JB JS Open Access 2020; 5(4). doi: 10.2106/jbjs.Oa.20.00107.

246. Tsukanaka M, Röhrl SM, von Schewelov T, Nordsletten L. Identification of femoral head center of bipolar hemiarthroplasy in radiostereometric analysis with elementary geometrical shape models. J Biomech 2016; 49(3): 469-73. doi: 10.1016/j.jbiomech.2015.11.054.

247. Turgeon TR, Gascoyne TC, Laende EK, Dunbar MJ, Bohm ER, Richardson CG. The assessment of the stability of the tibial component of a novel knee arthroplasty system using radiostereometric analysis. Bone Joint J 2018; 100-b(12): 1579-84. doi: 10.1302/0301-620x.100b12.Bjj-2018-0566.R1.

248. Turgeon TR, Hedden DR, Bohm ER, Burnell CD. Radiostereometric analysis and clinical outcomes of a novel reverse total hip system at two years. Bone Jt Open 2023; 4(5): 385-92. doi: 10.1302/2633-1462.45.Bjo-2023-0018.R1.

249. Turgeon TR, Righolt CH, Burnell CD, Gascoyne TC, Hedden DR, Bohm ER. Comparison of two hydroxyapatite-coated femoral components: a randomized clinical trial using radiostereometric analysis. Bone Joint J 2023; 105-b(10): 1045-51. doi: 10.1302/0301-620x.105b10.Bjj-2023-0427.R1.

250. Turgeon TR, Vasarhelyi E, Howard J, Teeter M, Righolt CH, Gascoyne T, et al. Randomized controlled trial comparing traditional versus enhanced-fixation designs of a novel cemented total knee arthroplasty tibial component. Bone Jt Open 2024; 5(1): 20-7. doi: 10.1302/2633-1462.51.Bjo-2023-0121.

251. Van de Kleut ML, Yuan X, Athwal GS, Teeter MG. Are short press-fit stems comparable to standard-length cemented stems in reverse shoulder arthroplasty? A prospective, randomized clinical trial. J Shoulder Elbow Surg 2022; 31(3): 580-90. doi: 10.1016/j.jse.2021.11.005.

252. Van de Kleut ML, Yuan X, Teeter MG, Athwal GS. Bony increased-offset reverse shoulder arthroplasty vs. metal augments in reverse shoulder arthroplasty: a prospective, randomized clinical trial with 2-year follow-up. J Shoulder Elbow Surg 2022; 31(3): 591-600. doi: 10.1016/j.jse.2021.11.007.

253. van der Lelij TJN, Marang-van de Mheen PJ, Kaptein BL, Koster LA, Ljung P, Nelissen R, et al. Migration and clinical outcomes of a novel cementless hydroxyapatite-coated titanium acetabular shell: two-year follow-up of a randomized controlled trial using radiostereometric analysis. Bone Joint J 2024; 106-b(2): 136-43. doi: 10.1302/0301-620x.106b2.Bjj-2023-0862.R1.

254. van der Lelij TJN, Marang-van de Mheen PJ, Kaptein BL, Toksvig-Larsen S, Nelissen R. Continued Stabilization of a Cementless 3D-Printed Total Knee Arthroplasty: Five-Year Results of a Randomized Controlled Trial Using Radiostereometric Analysis. J Bone Joint Surg Am 2023; 105(21): 1686-94. doi: 10.2106/jbjs.23.00221.

255. Van Der Voort P, ML DKN, Valstar ER, Kaptein BL, Fiocco M, R GHHN. Long-term migration of a cementless stem with different bioactive coatings. Data from a "prime" RSA study: lessons learned. Acta Orthop 2020; 91(6): 660-8. doi: 10.1080/17453674.2020.1840021.

256. van der Voort P, Valstar ER, Kaptein BL, Fiocco M, van der Heide HJ, Nelissen RG. Comparison of femoral component migration between Refobacin bone cement R and Palacos R + G in cemented total hip arthroplasty: A randomised controlled roentgen stereophotogrammetric analysis and clinical study. Bone Joint J 2016; 98-b(10): 1333-41. doi: 10.1302/0301-620x.98b10.37116.

257. van der Voort P, van Delft D, Valstar ER, Kaptein BL, Fiocco M, Nelissen RG. Migration behaviour of 2 clinically excellent cementless stems with different design rationales: 5-year follow-up of a randomised RSA-study. Hip Int 2022; 32(6): 747-58. doi: 10.1177/1120700021995482.

258. van Hamersveld KT, Marang-van de Mheen PJ, Koster LA, Nelissen R, Toksvig-Larsen S, Kaptein BL. Marker-based versus model-based radiostereometric analysis of total knee arthroplasty migration: a reanalysis with comparable mean outcomes despite distinct types of measurement error. Acta Orthop 2019; 90(4): 366-72. doi: 10.1080/17453674.2019.1605692.

259. van Hamersveld KT, Marang-van de Mheen PJ, Nelissen R. The Effect of Coronal Alignment on Tibial Component Migration Following Total Knee Arthroplasty: A Cohort Study with Long-Term Radiostereometric Analysis Results. J Bone Joint Surg Am 2019; 101(13): 1203-12. doi: 10.2106/jbjs.18.00691.

260. Van Hamersveld KT, Marang-Van De Mheen PJ, Nelissen R, Toksvig-Larsen S. Migration of all-polyethylene compared with metal-backed tibial components in cemented total knee arthroplasty. Acta Orthop 2018; 89(4): 412-7. doi: 10.1080/17453674.2018.1464317.

261. Van Hamersveld KT, Marang-Van De Mheen PJ, Nelissen R, Toksvig-Larsen S. Peri-apatite coating decreases uncemented tibial component migration: long-term RSA results of a randomized controlled trial and limitations of short-term results. Acta Orthop 2018; 89(4): 425-30. doi: 10.1080/17453674.2018.1469223.

262. van Hamersveld KT, Marang-van de Mheen PJ, Tsonaka R, Nilsson KG, Toksvig-Larsen S, Nelissen R. Risk Factors for Tibial Component Loosening: A Meta-Analysis of Long-Term Follow-up Radiostereometric Analysis Data. J Bone Joint Surg Am 2021; 103(12): 1115-24. doi: 10.2106/jbjs.20.01454.

263. van Hamersveld KT, Marang-van de Mheen PJ, Tsonaka R, Valstar ER, Toksvig-Larsen S. Fixation and clinical outcome of uncemented peri-apatite-coated versus cemented total knee arthroplasty : five-year follow-up of a randomised controlled trial using radiostereometric analysis (RSA). Bone Joint J 2017; 99-b(11): 1467-76. doi: 10.1302/0301-620x.99b11.Bjj-2016-1347.R3.

264. Van Hamersveld KT, Marang-Van De Mheen PJ, Van Der Heide HJL, Van Der Linden-Van Der Zwaag HMJ, Valstar ER, Nelissen R. Migration and clinical outcome of mobile-bearing versus fixed-bearing single-radius total knee arthroplasty. Acta Orthop 2018; 89(2): 190-6. doi: 10.1080/17453674.2018.1429108.

265. van Hooff ML, Heesterbeek PJC, Spruit M. Mechanical Stability of the Prodisc-C Vivo Cervical Disc Arthroplasty: A Preliminary, Observational Study Using Radiostereometric Analysis. Global Spine J 2020; 10(3): 294-302. doi: 10.1177/2192568219850763.

266. Van Laarhoven SN, Te Molder MEM, Van Hellemondt GG, Heesterbeek PJC. Acceptable migration of a fully cemented rotating hinge-type knee revision system measured in 20 patients with model-based RSA with a 2-year follow-up. Acta Orthop 2023; 94: 185-90. doi: 10.2340/17453674.2023.12305.

267. van Ooij B, Sierevelt IN, van der Vis HM, Hoornenborg D, Haverkamp D. What is the role of cemented fixation in total knee arthroplasty? The two-year results of a randomized RSA controlled trial. Bone Joint J 2021; 103-b(1): 98-104. doi: 10.1302/0301-620x.103b1.Bjj-2020-0788.R1.

268. Vind TD, Jørgensen PB, Vainorius D, Jakobsen SS, Søballe K, Stilling M. Migration pattern of cemented Exeter short stem in Dorr type A femurs. A prospective radiostereometry study with 2-year follow-up. Arch Orthop Trauma Surg 2023; 143(2): 1071-80. doi: 10.1007/s00402-021-04307-y.

269. von Schewelov T, Carlsson A, Sanzén L, Besjakov J. Continuous distal migration and internal rotation of the C-stem prosthesis without any adverse clinical effects: an RSA study of 33 primary total hip arthroplasties followed for up to ten years. Bone Joint J 2014; 96-b(5): 604-8. doi: 10.1302/0301-620x.96b5.33580.

270. Weber E, Flivik C, Sundberg M, Flivik G. Migration pattern of a short uncemented stem with or without collar: a randomised RSA-study with 2 years follow-up. Hip Int 2021; 31(4): 500-6. doi: 10.1177/1120700019888471.

271. Weber E, Olsson C, Kesteris U, Flivik G. Is a hollow centralizer necessary when using a polished, tapered, cemented femoral stem? Acta Orthop 2017; 88(4): 377-82. doi: 10.1080/17453674.2017.1315553.

272. Weber E, Sundberg M, Flivik G. Design modifications of the uncemented Furlong hip stem result in minor early subsidence but do not affect further stability: a randomized controlled RSA study with 5-year follow-up. Acta Orthop 2014; 85(6): 556-61. doi: 10.3109/17453674.2014.958810.

273. Wierer T, Forst R, Mueller LA, Sesselmann S. Radiostereometric migration analysis of the Lubinus SP II hip stem: 59 hips followed for 2 years. Biomed Tech (Berl) 2013; 58(4): 333-41. doi: 10.1515/bmt-2012-0038.

274. Williams HA, Broberg JS, Howard JL, Lanting BA, Teeter MG. Effect of gap balancing and measured resection techniques on implant migration and contact kinematics of a cementless total knee arthroplasty. Knee 2021; 31: 86-96. doi: 10.1016/j.knee.2021.05.011.

275. Wilson DA, Hubley-Kozey CL, Astephen Wilson JL, Dunbar MJ. Pre-operative muscle activation patterns during walking are associated with TKA tibial implant migration. Clin Biomech (Bristol, Avon) 2012; 27(9): 936-42. doi: 10.1016/j.clinbiomech.2012.06.012.

276. Wilson DA, Richardson G, Hennigar AW, Dunbar MJ. Continued stabilization of trabecular metal tibial monoblock total knee arthroplasty components at 5 years-measured with radiostereometric analysis. Acta Orthop 2012; 83(1): 36-40. doi: 10.3109/17453674.2011.645196.

277. Winther NS, Jensen CL, Jensen CM, Lind T, Schrøder HM, Flivik G, et al. Comparison of a novel porous titanium construct (Regenerex®) to a well proven porous coated tibial surface in cementless total knee arthroplasty - A prospective randomized RSA study with two-year follow-up. Knee 2016; 23(6): 1002-11. doi: 10.1016/j.knee.2016.09.010.

278. Wojtowicz R, Henricson A, Nilsson KG, Crnalic S. Uncemented monoblock trabecular metal posterior stabilized high-flex total knee arthroplasty: similar pattern of migration to the cruciate-retaining design - a prospective radiostereometric analysis (RSA) and clinical evaluation of 40 patients (49 knees) 60 years or younger with 9 years' follow-up. Acta Orthop 2019; 90(5): 460-6. doi: 10.1080/17453674.2019.1626097.

279. Wolf O, Mattsson P, Milbrink J, Larsson S, Mallmin H. The effects of different weight-bearing regimes on press-fit cup stability: a randomised study with five years of follow-up using radiostereometry. Int Orthop 2012; 36(4): 735-40. doi: 10.1007/s00264-011-1413-5.

280. Wolterbeek N, Garling EH, Mertens BJ, Nelissen RG, Valstar ER. Kinematics and early migration in single-radius mobile- and fixed-bearing total knee prostheses. Clin Biomech (Bristol, Avon) 2012; 27(4): 398-402. doi: 10.1016/j.clinbiomech.2011.10.013.

281. Xu J, Cao H, Sesselmann S, Taylor D, Forst R, Seehaus F. Article. Model-Based Roentgen Stereophotogrammetric Analysis Using Elementary Geometrical Shape Models: Reliability of Migration Measurements for an Anatomically Shaped Femoral Stem Component. Appl Sci-Basel 2020; 10(23): 13. doi: 10.3390/app10238507.

282. Yilmaz M, Holm CE, Lind T, Flivik G, Odgaard A, Petersen MM. Bone remodeling and implant migration of uncemented femoral and cemented asymmetrical tibial components in total knee arthroplasty - DXA and RSA evaluation with 2-year follow up. Knee Surg Relat Res 2021; 33(1): 25. doi: 10.1186/s43019-021-00111-5.

283. Yüksel Y, Koster LA, Kaptein BL, Nelissen R, den Hollander P. No difference in component migration at five years between the cemented cruciate-retaining ATTUNE and PFC-Sigma knee prosthesis: an update of a randomized clinical radiostereometry trial. Bone Joint J 2023; 105-b(11): 1168-76. doi: 10.1302/0301-620x.105b11.Bjj-2022-0839.R4.

284. Zampelis V, Belfrage O, Tägil M, Sundberg M, Flivik G. Decreased migration with locally administered bisphosphonate in cemented cup revisions using impaction bone grafting technique. Acta Orthop 2018; 89(1): 17-22. doi: 10.1080/17453674.2017.1371468.

285. Zampelis V, Flivik G, Kesteris U. No effect of femoral canal jet-lavage on the stability of cementless stems in primary hip arthroplasty: a randomised RSA study with 6 years follow-up. Hip Int 2020; 30(4): 417-22. doi: 10.1177/1120700019843123.
