# Supplementary material for: Adherence to the RSA and CT-RSA guideline items in clinical prosthesis migration studies: a systematic review
Source: Acta Orthop. 2025 May 27;96:380–6. doi: 10.2340/17453674.2025.43750 (PMC12118374; doi:10.2340/17453674.2025.43750)
Supplement: Supplementary file 2 [file ActaO-96-43750-s2.pdf]

Appendix 2. Search strategy

PubMed
(("Radiostereometric Analysis"[Mesh] OR "Photogrammetry"[mesh] OR "RSA"[tiab] OR "Radiostereometric Analysis"[tw] OR "Radiostereometry"[tw] OR "Radiostereometric"[tw] OR "Radiostereometr*"[tw] OR "Radio stereometry"[tw] OR "Radio stereometr*"[tw] OR "roentgen stereophotogrammetric analysis"[tw] OR "Stereophotogrammetry"[tw] OR "Stereophotogrammetric"[tw] OR "Stereophotogrammetr*"[tw] OR "Stereo photogrammetry"[tw] OR "Stereo photogrammetric"[tw] OR "Stereo photogrammetr*"[tw] OR "Rontgen Stereophotogrammetr*"[tw] OR "Roentgen Stereometry"[tw] OR "Roentgen Stereometr*"[tw] OR "Photofluorography"[tw] OR "roentgen fluoroscopic"[tw] OR "roentgen fluoroscopy"[tw] OR "roentgen fluoroscop*"[tw]) AND ("Arthroplasty, replacement"[Mesh] OR "Joint Prosthesis"[Mesh] OR "Arthroplast*"[tw] OR "Hemiarthroplast*"[tw] OR "Hemi-arthroplast*"[tw] OR "Prosthesis"[tw] OR "Prostheses"[tw] OR "endoprosthesis"[tw] OR "endoprostheses"[tw] OR "Replacement"[tw] OR "reconstruction"[tw] OR "resurfacing"[tw] OR "resurfaced"[tw] OR "Implant"[tw] OR "Implants"[tw]) AND ("2012/01/01"[PDAT] : "3000/12/31"[PDAT]))

Embase
((exp *"Radiostereometric Analysis"/ OR exp *"Photogrammetry"/ OR "RSA".ti,ab OR "Radiostereometric Analysis".ti,ab OR "Radiostereometry".ti,ab OR "Radiostereometric".ti,ab OR "Radiostereometr*".ti,ab OR "Radio stereometry".ti,ab OR "Radio stereometr*".ti,ab OR "roentgen stereophotogrammetric analysis".ti,ab OR "Stereophotogrammetry".ti,ab OR "Stereophotogrammetric".ti,ab OR "Stereophotogrammetr*".ti,ab OR "Stereo photogrammetry".ti,ab OR "Stereo photogrammetric".ti,ab OR "Stereo photogrammetr*".ti,ab OR "Rontgen Stereophotogrammetr*".ti,ab OR "Roentgen Stereometry".ti,ab OR "Roentgen Stereometr*".ti,ab OR "Photofluorography".ti,ab OR "roentgen fluoroscopic".ti,ab OR "roentgen fluoroscopy".ti,ab OR "roentgen fluoroscop*".ti,ab) AND (exp *"orthopedic prostheses, orthoses and implants"/ OR exp *"Arthroplasty"/ OR "Arthroplast*".ti,ab OR "Hemiarthroplast*".ti,ab OR "Hemi-arthroplast*".ti,ab OR "Prosthesis".ti,ab OR "Prostheses".ti,ab OR "endoprosthesis".ti,ab OR "endoprostheses".ti,ab OR "Replacement".ti,ab OR "reconstruction".ti,ab OR "resurfacing".ti,ab OR "resurfaced".ti,ab OR "Implant".ti,ab OR "Implants".ti,ab) AND 2012:2024.(sa_year) NOT (conference review or conference abstract).pt)

Web of Science
((TI=("Radiostereometric Analysis" OR "Photogrammetry" OR "RSA" OR "Radiostereometric Analysis" OR "Radiostereometry" OR "Radiostereometric" OR "Radiostereometr*" OR "Radio stereometry" OR "Radio stereometr*" OR "roentgen stereophotogrammetric analysis" OR "Stereophotogrammetry" OR "Stereophotogrammetric" OR "Stereophotogrammetr*" OR "Stereo photogrammetry" OR "Stereo photogrammetric" OR "Stereo photogrammetr*" OR "Rontgen Stereophotogrammetr*" OR "Roentgen Stereometry" OR "Roentgen Stereometr*" OR "Photofluorography" OR "roentgen fluoroscopic" OR "roentgen fluoroscopy" OR "roentgen fluoroscop*") OR AK=("Radiostereometric Analysis" OR "Photogrammetry" OR "RSA" OR "Radiostereometric Analysis" OR "Radiostereometry" OR "Radiostereometric" OR "Radiostereometr*" OR "Radio stereometry" OR "Radio stereometr*" OR "roentgen stereophotogrammetric analysis" OR "Stereophotogrammetry" OR "Stereophotogrammetric" OR "Stereophotogrammetr*" OR "Stereo photogrammetry" OR "Stereo photogrammetric" OR "Stereo photogrammetr*" OR "Rontgen Stereophotogrammetr*" OR "Roentgen Stereometry" OR "Roentgen Stereometr*" OR

"Photofluorography" OR "roentgen fluoroscopic" OR "roentgen fluoroscopy" OR "roentgen fluoroscop*") OR AB=("Radiostereometric Analysis" OR "Photogrammetry" OR "RSA" OR "Radiostereometric Analysis" OR "Radiostereometry" OR "Radiostereometric" OR "Radiostereometr*" OR "Radio stereometry" OR "Radio stereometr*" OR "roentgen stereophotogrammetric analysis" OR "Stereophotogrammetry" OR "Stereophotogrammetric" OR "Stereophotogrammetr*" OR "Stereo photogrammetry" OR "Stereo photogrammetric" OR "Stereo photogrammetr*" OR "Rontgen Stereophotogrammetr*" OR "Roentgen Stereometry" OR "Roentgen Stereometr*" OR "Photofluorography" OR "roentgen fluoroscopic" OR "roentgen fluoroscopy" OR "roentgen fluoroscop*")) AND (TI=("Arthroplasty" OR "Arthroplast*" OR "Hemiarthroplast*" OR "Hemi-arthroplast*" OR "Prosthesis" OR "Prostheses" OR "endoprosthesis" OR "endoprostheses" OR "Replacement" OR "resurfacing" OR "resurfaced" OR "Implant" OR "Implants") OR AK=("Arthroplasty" OR "Arthroplast*" OR "Hemiarthroplast*" OR "Hemi-arthroplast*" OR "Prosthesis" OR "Prostheses" OR "endoprosthesis" OR "endoprostheses" OR "Replacement" OR "resurfacing" OR "resurfaced" OR "Implant" OR "Implants") OR AB=("Arthroplasty" OR "Arthroplast*" OR "Hemiarthroplast*" OR "Hemi-arthroplast*" OR "Prosthesis" OR "Prostheses" OR "endoprosthesis" OR "endoprostheses" OR "Replacement" OR "resurfacing" OR "resurfaced" OR "Implant" OR "Implants")) AND PY=(2012 OR 2013 OR 2014 OR 2015 OR 2016 OR 2017 OR 2018 OR 2019 OR 2020 OR 2021 OR 2022 OR 2023 OR 2024) NOT DT=("meeting abstract"))

Cochrane
(("Radiostereometric Analysis" OR "Photogrammetry" OR "RSA" OR "Radiostereometric Analysis" OR "Radiostereometry" OR "Radiostereometric" OR "Radiostereometr*" OR "Radio stereometry" OR "Radio stereometr*" OR "roentgen stereophotogrammetric analysis" OR "Stereophotogrammetry" OR "Stereophotogrammetric" OR "Stereophotogrammetr*" OR "Stereo photogrammetry" OR "Stereo photogrammetric" OR "Stereo photogrammetr*" OR "Rontgen Stereophotogrammetr*" OR "Roentgen Stereometry" OR "Roentgen Stereometr*" OR "Photofluorography" OR "roentgen fluoroscopic" OR "roentgen fluoroscopy" OR "roentgen fluoroscop*"):ti,ab,kw AND ("Arthroplasty" OR "Arthroplast*" OR "Hemiarthroplast*" OR "Hemi-arthroplast*" OR "Prosthesis" OR "Prostheses" OR "endoprosthesis" OR "endoprostheses" OR "Replacement" OR "reconstruction" OR "resurfacing" OR "resurfaced" OR "Implant" OR "Implants"):ti,ab,kw) NOT DT=("meeting abstract") AND PY=(2012 OR 2013 OR 2014 OR 2015 OR 2016 OR 2017 OR 2018 OR 2019 OR 2020 OR 2021 OR 2022 OR 2023 OR 2024)

Emcare
((exp *"Radiostereometric Analysis"/ OR exp *"Photogrammetry"/ OR "RSA".ti,ab OR "Radiostereometric Analysis".ti,ab OR "Radiostereometry".ti,ab OR "Radiostereometric".ti,ab OR "Radiostereometr*".ti,ab OR "Radio stereometry".ti,ab OR "Radio stereometr*".ti,ab OR "roentgen stereophotogrammetric analysis".ti,ab OR "Stereophotogrammetry".ti,ab OR "Stereophotogrammetric".ti,ab OR "Stereophotogrammetr*".ti,ab OR "Stereo photogrammetry".ti,ab OR "Stereo photogrammetric".ti,ab OR "Stereo photogrammetr*".ti,ab OR "Rontgen Stereophotogrammetr*".ti,ab OR "Roentgen Stereometry".ti,ab OR "Roentgen Stereometr*".ti,ab OR "Photofluorography".ti,ab OR "roentgen fluoroscopic".ti,ab OR "roentgen fluoroscopy".ti,ab OR "roentgen fluoroscop*".ti,ab) AND (exp *"orthopedic prostheses, orthoses and implants"/ OR exp *"Arthroplasty"/ OR "Arthroplast*".ti,ab OR "Hemiarthroplast*".ti,ab OR "Hemi-arthroplast*".ti,ab OR "Prosthesis".ti,ab OR "Prostheses".ti,ab OR "endoprosthesis".ti,ab OR "endoprostheses".ti,ab OR

"Replacement".ti,ab OR "reconstruction".ti,ab OR "resurfacing".ti,ab OR "resurfaced".ti,ab OR "Implant".ti,ab OR "Implants".ti,ab) AND 2012:2024.(sa_year))

Academic Search Premier

((TI("Radiostereometric Analysis" OR "Photogrammetry" OR "RSA" OR "Radiostereometric Analysis" OR "Radiostereometry" OR "Radiostereometric" OR "Radiostereometr*" OR "Radio stereometry" OR "Radio stereometr*" OR "roentgen stereophotogrammetric analysis" OR "Stereophotogrammetry" OR "Stereophotogrammetric" OR "Stereophotogrammetr*" OR "Stereo photogrammetry" OR "Stereo photogrammetric" OR "Stereo photogrammetr*" OR "Rontgen Stereophotogrammetr*" OR "Roentgen Stereometry" OR "Roentgen Stereometr*" OR "Photofluorography" OR "roentgen fluoroscopic" OR "roentgen fluoroscopy" OR "roentgen fluoroscop*") OR KW("Radiostereometric Analysis" OR "Photogrammetry" OR "RSA" OR "Radiostereometric Analysis" OR "Radiostereometry" OR "Radiostereometric" OR "Radiostereometr*" OR "Radio stereometry" OR "Radio stereometr*" OR "roentgen stereophotogrammetric analysis" OR "Stereophotogrammetry" OR "Stereophotogrammetric" OR "Stereophotogrammetr*" OR "Stereo photogrammetry" OR "Stereo photogrammetric" OR "Stereo photogrammetr*" OR "Rontgen Stereophotogrammetr*" OR "Roentgen Stereometry" OR "Roentgen Stereometr*" OR "Photofluorography" OR "roentgen fluoroscopic" OR "roentgen fluoroscopy" OR "roentgen fluoroscop*") OR AB("Radiostereometric Analysis" OR "Photogrammetry" OR "RSA" OR "Radiostereometric Analysis" OR "Radiostereometry" OR "Radiostereometric" OR "Radiostereometr*" OR "Radio stereometry" OR "Radio stereometr*" OR "roentgen stereophotogrammetric analysis" OR "Stereophotogrammetry" OR "Stereophotogrammetric" OR "Stereophotogrammetr*" OR "Stereo photogrammetry" OR "Stereo photogrammetric" OR "Stereo photogrammetr*" OR "Rontgen Stereophotogrammetr*" OR "Roentgen Stereometry" OR "Roentgen Stereometr*" OR "Photofluorography" OR "roentgen fluoroscopic" OR "roentgen fluoroscopy" OR "roentgen fluoroscop*")) AND (TI("Arthroplasty" OR "Arthroplast*" OR "Hemiarthroplast*" OR "Hemi-arthroplast*" OR "Prosthesis" OR "Prostheses" OR "endoprosthesis" OR "endoprostheses" OR "Replacement" OR "resurfacing" OR "resurfaced" OR "Implant" OR "Implants") OR KW("Arthroplasty" OR "Arthroplast*" OR "Hemiarthroplast*" OR "Hemi-arthroplast*" OR "Prosthesis" OR "Prostheses" OR "endoprosthesis" OR "endoprostheses" OR "Replacement" OR "resurfacing" OR "resurfaced" OR "Implant" OR "Implants") OR AB("Arthroplasty" OR "Arthroplast*" OR "Hemiarthroplast*" OR "Hemi-arthroplast*" OR "Prosthesis" OR "Prostheses" OR "endoprosthesis" OR "endoprostheses" OR "Replacement" OR "resurfacing" OR "resurfaced" OR "Implant" OR "Implants")) AND PY=(2012 OR 2013 OR 2014 OR 2015 OR 2016 OR 2017 OR 2018 OR 2019 OR 2020 OR 2021 OR 2022 OR 2023 OR 2024 NOT DT=("meeting abstract"))
